# Transcriptome Analysis of the Effects of Shell Removal and Exogenous Gibberellin on Germination of Zanthoxylum Seeds

**DOI:** 10.1038/s41598-017-07424-0

**Published:** 2017-08-17

**Authors:** Jikang Sun, Ping Wang, Tao Zhou, Jian Rong, Hao Jia, Zhiming Liu

**Affiliations:** 1 College of Life Science and Technology, Central South University of Forestry and Technology, Changsha, Hunan China; 2 College of Environmental Science and Engineering, Central South University of Forestry and Technology, Changsha, Hunan China; 3 Department of Biology, Eastern New Mexico University, Portales, NM88130 USA

## Abstract

The zanthoxylum seeds are oil-rich and have a very thick, dense and oily shell. In the natural conditions the seeds have a very low germination rate. Prior to treatment with GAs to promote germination, the seeds were usually soaked in sulfuric acid to remove shells easily. A high-throughput sequencing of mRNAs was performed to investigate the effects of the above treatments on the germination of zanthoxylum seeds. Seven libraries were assembled into 100,982 unigenes and 59,509 unigenes were annotated. We focused on the expression profiles of the key genes related to the oil metabolisms and hormone regulations during seed germination. Our data indicated the endogenous ABA of seeds was rich. The effects that the exogenous GAs promoted germination were apparent in the secong day of germination. Especially, for the first time our results indicated the exogenous GAs lowered the aerobic metabolism including the oil metabolisms during imbibition. We inferred that the exogenous GAs had inhibitory effects on the oil metabolisms to avoide oxidative damages to the imbibed seeds, and the seed shell played the role similiar to the exogenous GAs in the initial stage of germination in the natural conditions.

## Introduction

*Zanthoxylum dissitum* Hemsl.(zanthoxylum), a commonly used medicinal plant in China, usually grows in the mountainous region of 500 ~ 1200 meters above sea level in southern China. Currently the natural resource of zanthoxylum has become diminished because of excessive utilization. Further, zanthoxylum is difficult to cultivate and has a very low germination rate in the natural conditions^1^. Under laboratory conditions, the seeds of zanthoxylum seldom germinated and were usually soaked in 80% sulfuric acid for three minutes to remove shells easily before treated by gibberellins (GAs) to promote germination.

Germination incorporates the processes that begin with the uptake of water by the quiescent dry seeds, and finish with the elongation of the embryonic axis^2^. Triacylglycerides (TAG) are usually stored in the oil body of seeds of plants. Generally, TAG mobilization provides germinating seeds with both carbon skeletons and energy^3, 4^. The β-oxidation of fatty acid involves reserve mobilization and developmental signaling during germination^5–7^. The β-oxidation generates Hydrogen peroxide (H_2_O_2_), NADH and acetyl-CoA. Not only is H_2_O_2_ potentially damaging as a result of oxidation reactions, but also is a potential signaling molecule involved in stress responses^8, 9^. H_2_O_2_ functions as a promoter of seed germination by oxidizing germination inhibitors and is vital for seed dormancy breaking during imbibition^10, 11^. ABA inhibits seed germination^12^, and GAs promote seed germination in many species^13, 14^. Evidence indicates that the antagonism between ABA and GAs plays a key role in controlling seed germination^15^. For example, GAs induce transcription of α-amylase in the aleurone layer of cereal seeds which is significantly suppressed by ABA^16, 17^. Brassinosteroids (BRs) function in growth and developmental events, including cell elongation, seed germination, and etc^18, 19^. BRs generally counteract ABA on root growth, seed germination, and possibly stomatal movement^20^. Jasmonates (JAs) are essential signaling molecules modulating the plant response to biotic and abiotic stresses as well as several growth and developmental traits^21, 22^. JAs signaling antagonizes GA-mediated reduction of the DELLA protein^23^. ABA and JAs have synergistic effects on seed germination, and seedling cotyledon expansion and establishment^24^. The phenilpropanoid metabolism is another defensive mechanism^25^. Some phenylpropanoids can polymerize to form defensive structures, such as lignin^26^.

Our previous research showed that the germination rate of the GAs-treated seeds with shells removed was above 80%, but the water-treated seeds usually failed to germinate and decayed in the third day of germination. In this study, we constructed seven libraries including C0 (the seeds cold-stratificated for three months and shells removed) group, W1 (water-treated seed germinating for one day) group, W2 (water-treated seed germinating for two days, two biological repeats, W2.1, W2.2) group, GA1 (GAs-treated seed germinating for one day) group, GA2 (GAs-treated seed germinating for two days, two biological repeats, GA2.1, GA2.2) group. A high-throughput sequencing of mRNAs was performed. The expressions of the genes associated with early stages of germination were investigated to reveal the effects of the above treatments on the seed germination.

## Results

### The morphology in the early stage of germination of zanthoxylum seeds

**Figure 1:**
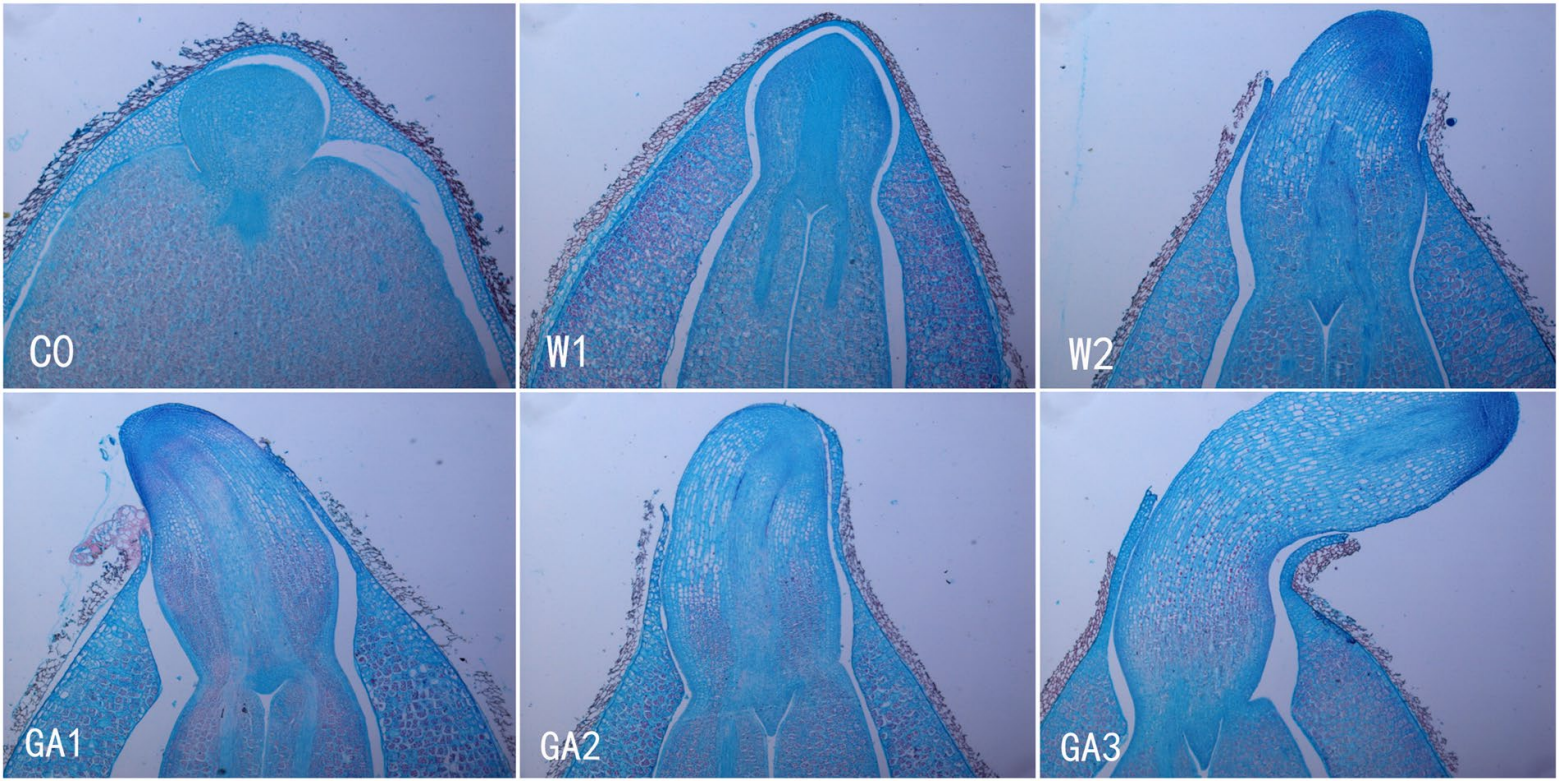
The morphology during the early stage of germination of zanthoxylum seeds.

The seeds of zanthoxylum were collected in Zhangjiajie, Hunan province in December 2015. The newly collected seeds were cold-stratificated for three months. After the shells were removed, the seeds were treated by GAs and distilled water before incubation. Our research showed that the water-treated seeds usually decayed in the third day of germination, while the GAs-treated seeds could continue to germinate. The results showed that there were few differences in the morphology between the water-treated seeds and GAs-treated seeds during the first and second day of germination. The radicle and hypocotyl of the GAs-treated seed germinating for three days broke through the seed coat, and had a visible stretch after germinating for three days (Fig. 1).

### Transcriptome sequencing and de novo assembly

The raw reads sequenced using RNA-Seq technique were further filtered by removing adaptors, ambiguous nucleotides and low-quality sequences to generate clean reads. After trimming the raw reads, there were 42.90 million, 44.00 million, 44.20 million, 39.69 million, 48.01 million, 43.06 million and 48.27 million clean reads generated from C0, W1, W2.1, W2.2, GA1, GA2.1 and GA2.2, respectively (Table 1).

**Table 1:** Overview of the sequencing reads.

Samples	C0	W1	W2.1	W2.2	GA1	GA2.1	GA2.2
Raw read number	43820348	44961900	45226620	40802038	49135056	44266652	49428050
Clean read number	42905020	44009068	44208072	39698792	48016010	43065538	48274642
Raw read base pairs	5477543500	5620237500	5653327500	5100254750	6141882000	5533331500	6178506250
Clean read base	5363127500	5501133500	5526009000	4962349000	6002001250	5383192250	6034330250
Q20%	96.20%	96.15%	96.19%	95.78%	96.16%	95.74%	96.00%
Q30%	92.27%	92.22%	92.25%	91.43%	92.26%	91.41%	91.93%
GC%	45.97%	44.76%	46.83%	47.49%	44.76%	44.72%	44.83%

Q20: The percentage of bases with quality value larger than 20. Q30: The percentage of bases with quality value larger than 30. GC%: The percentage of proportion of guanidine and cytosine nucleotides among total nucleotides.

The clean reads obtained from the seven different transcriptome libraries were pooled and assembled using Trinity software. 100,982 unigenes were achieved with an average length of 636.41 base pairs and N50 of 944 bp (Table S1). The size distribution of the unigenes was shown in Figure S1. All unigenes provided a sequence basis for analysis of gene expression during the early stage of germination of zanthoxylum seeds.

### Unigene annotation to public database

**Figure 2:**
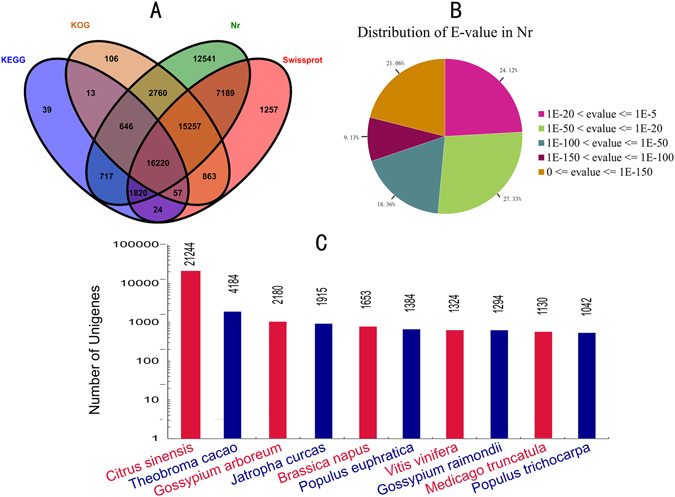
Characteristics of homology search of zanthoxylum unigenes. (**A**) Venn diagram of number of unigenes annotated by BLASTx with a cut-off E-value 1e^−05^ against protein databases. Numbers in the circles indicate the number of unigenes annotated by single or multiple databases; (**B**) E-value distribution of the top BLASTx hits against the nr database; (**C**) Number of unigenes matching the 20 top species using BLASTx in the nr database.

56.59% (57,150/100,982) of unigenes could be annotated using the NCBI nr database, while 42,687 were annotated using the Swiss-Prot protein database. In addition, 19,536 and 35,922 unigenes could be annotated according to the Kyoto Encyclopedia of Genes and Genomes (KEGG) and Cluster of Orthologous Groups of protein (KOG) database, respectively. About 16.06% (16,220/100,982) of the unigenes had hits in all four databases (Fig. 2A). Based on the NCBI nr database, 75.87% of those showed strong homology (E-value < 1e^−20^) to available plant sequences (Fig. 2B). As shown in Fig. 2C, 31,176 unigenes were annotated to 5 top-hit species. It was notable that 21,244 unigenes were annotated to *Citrus sinensis*, zanthoxylum and *Citrus sinensis* belonged to the rutaceae plants.

GO assignment was performed to classify functions of the predicted zanthoxylum genes (Figure S2). KEGG pathway-based analysis helps to identify the biological pathways that are related to unigenes. In the top 20 annotation KEGG pathways, the pathways including metabolic pathways, ribosome, RNA transport, spliceosome, plant-pathogen interaction and plant hormone signal transduction were worth noting (Table S2). To classify orthologous proteins, the assembled unigenes were compared against KOG. 35922 unigenes were clustered into 25 categories (Figure S3)

### Identification of differentially expressed genes (DEGs)

To assess the normality of the RNA-Seq data in the seven digital gene expression tag (DGE) libraries, we calculated the distribution of unique reads in each DGE libraries (Figure S4). This value was the ratio of the number of bases in a gene covered by unique mapping reads to the total bases in that from our transcriptome reference database. The distribution over different reads abundance categories showed similar patterns among all seven libraries. Above 60% of the sequences had a coverage of more than 80%. Next, we calculated the unigene expression using the uniquely mapped DGE fragments and normalized the results to fragments per kilobase million (FPKM). The results from the biological replicates (W2.1 vs W2.2, GA2.1 vs GA2.2) were highly similar (Figure S5), suggesting good reproducibility of the method. The DEGs during the course of seed germination were explored by using DEGseq with the criteria of |log2Ratio| ≥ 1 and FDR ≤ 0.01. In total, the number of DEGs between two consecutive stages was shown in Figure S6.

### PC analysis based on differentially expressed genes

**Figure 3:**
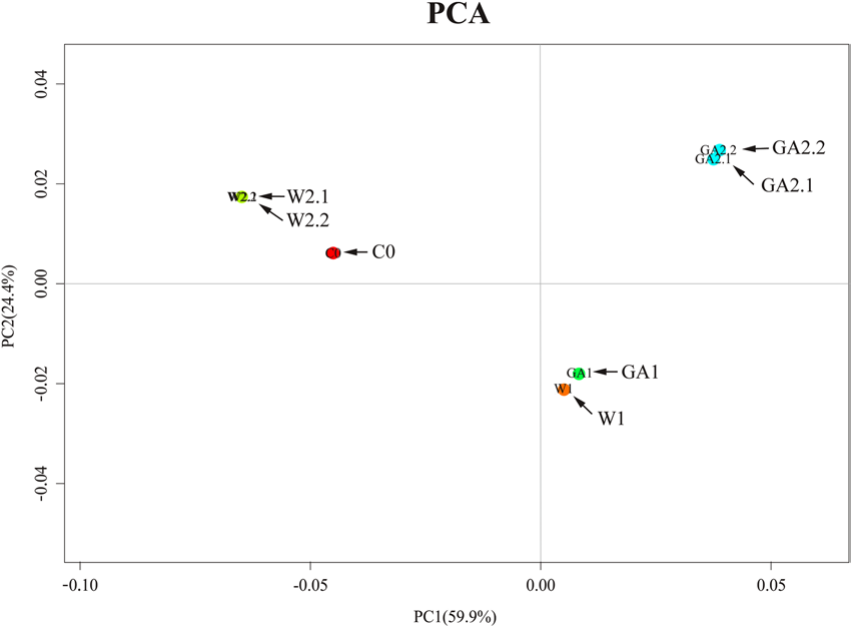
Principle component analysis of different germination stages based on the variation of the expression of differentially expressed unigenes.

We conducted a principle component analysis (PCA) on the gene expression data to summarize variation into the first two principal components to facilitate the comparison between the samples (Fig. 3). PC1 and PC2 represented 59.9% and 24.4% of total variability of unigenes expression, respectively. Interestingly, PC2 separated imbibition, as imbibed seeds (GA1, W1) appeared to have negative PC2 scores. However, PC1 separated germination and inhibited germination stages, as shown that germinating seeds (GA2.1, GA2.2) had positive PC1 scores while inhibited germination seeds (C0, W2.1, W2.2) had negative PC1 scores. W2 and C0 were clustered together, which suggested that the germination process was inhibited. W1 and GA1 were clustered together, which showed seeds were imbibed. The cluster of GA2 indicated that seeds germinated.

### Functional analysis of differentially expressed genes

To investigate the biological events that the DEGs are mainly involved during seed germination, GO term enrichments were conducted (Table S3). The results showed that the oxidation-reduction, metabolic process and response to stimulus and stress were related to various physiological functions associated with the seed germination of zanthoxylum.

KEGG pathways enrichment analysis of up-regulated and down-regulated unigenes during imbibition was performed, respectively (Table S4). The enrichment pathways of the up-regulated unigenes in the stage of C0-W1 involved many aerobic pathways including citrate cycle, glycolysis/gluconeogenesis, fatty acid metabolism, starch and sucrose metabolism, pyruvate metabolism, which differentiated from those in the stage of C0-GA1. The data indicated the aerobic metabolisms of the water-treated seeds were more active than the GAs-treated seeds during imbibition.

Considering oil-rich seeds of zanthoxylum, the oil metabolisms were further investigated. The *TAG lipase* (*UNIGENE0080821*, *UNIGENE0080823*) was highly expressed in C0/W1 seeds but lowly in the GAs-treated seeds. The expressions of the enzymes catalyzing initial reactions of β-oxidation of fatty acid including *long−chain acyl−CoA synthetase* (*UNIGENE0081657*, *UNIGENE0081658*), *acyl−CoA dehydrogenase* (*UNIGENE0064481*) and *enoyl−CoA hydratase 3−hydroxyacyl−CoA dehydrogenase* (*UNIGENE0078809*, *UNIGENE0078807*) were higher in W1 seeds than those in the GAs-treated seeds (Figure S7). The richer mRNA of the above key enzymes indicated more active TAG hydrolysis and β-oxidation of fatty acid during imbibition of W1 seeds. The acetyl-CoA derived from β-oxidation of fatty acid were decomposed thoroughly in citrate cycle pathways. The expressions of the enzymes catalyzing the initial two reactions of citrate cycle including *citrate synthase* (*UNIGENE0072817*, *UNIGENE0072090*) and *aconitate hydratase* (*UNIGENE0085001*, *UNIGENE0085007*, *UNIGENE0085011*) were higher in W1 seeds than those in the GAs-treated seeds (Figure S8). Our data strongly supported that more active oil metabolisms happened in the water-treated seeds, and also indicated that the exogenous GAs conduced to down-regulation of the oil metabolisms during imbibition.

We also focused on several important antioxidant enzymes, such as peroxidase (UNIGENE0037654), superoxide dismutase (UNIGENE0025883, UNIGENE0044110), catalase (UNIGENE0082845, UNIGENE0082846), L-ascorbate peroxidase (UNIGENE0022955, UNIGENE0059338), glutathione reductase (UNIGENE0067439). The data showed the expressions were higher in W1 seeds than those in the GAs-treated seeds (Figure S9). The higher expression of antioxidant enzymes was response to peroxide accumulation in defense to the oxidative damages caused by active aerobic metabolism in W1 seeds. Seed 1-cysteine peroxiredoxin antioxidants are not involved in dormancy, but contribute to inhibition of germination during stress^27^. Surprisingly, the expression pattern of 1-Cys peroxiredoxin (UNIGENE0031506) matched the seeds germination activity well. The expression of 1-Cys peroxiredoxin were the highest in C0/W2 seeds, but the lowest in GA2 seeds, which indicated the germination of W2 seeds was inhibited.

The enrichment pathways of the down-regulated unigenes in the stage of C0-GA1 and W1-GA1 involved RNA degradation pathway. The expressions of *CCR4−NOT transcription complex* (*UNIGENE0022101*, *UNIGENE0056383*, *UNIGENE0071262*) and *enhancer of mRNA−decapping protein* (*UNIGENE0027382*, *UNIGENE0085164*) were higher in W1 seeds than those in GA1 seeds. In contrast, the expression of *U6 snRNA−associated Sm−like protein LSm* (*UNIGENE0013171*, *UNIGENE0029527*, *UNIGENE0029526*) was higher in GA1 than that in W1 (Figure S10). The CCR4-NOT complex is one of the major cellular mRNA deadenylases and is linked to various cellular processes including bulk mRNA degradation, translational repression during translational initiation and general transcription regulation^28–30^. In the process of mRNA degradation, the enhancer of mRNA-decapping protein plays a role in mRNA decapping^31^. The U6 snRNA−associated Sm−like protein LSm plays a critical role in the regulation of development-related gene expression and is a component of LSM protein complexes which are essential for accurate splicing of selected development-related mRNAs through stabilization of the spliceosomal U6 snRNA^32, 33^. Our results indicated the mRNA degradation was less active but RNA processing was more active in GA1 seeds than those in W1 seeds, indicating GA1 seeds were in a favorable germination state.

### Dynamic expression profiles of DEGs

To get dynamic expression patterns of DEGs during seed germination, Short Time-series Expression Miner (STEM) software was performed to classify all the DEGs according to their abundance changes. The 42,453 DEGs were classified into 8 clusters according to their expression patterns (Figure S11). Six significant expression profiles (profile 1, profile 2, profile 3, profile 4, profile 5, profile 6) were identified. As shown in Figure S11, significantly different profiles were represented by different background colors. During the germination of the water-treated seeds, the most abundant group was profile 5, with 9741 genes whose expression showed a positive-negative slope. During the germination of the GAs-treated seeds, the most abundant group was profile 6, with 8896 genes whose expression showed a positive-zero slope.

Profile 5 and profile 6 were the two represented patterns. The GO functional analysis of profile 5 (water-treated seeds) and profile 6 (GAs-treated seeds) were listed in Table S5. The comparison of above GO functional analysis suggested carboxylic acid metabolism, oxoacid metabolism, organic acid metabolism and oxidoreductase activity were important in the early stage of germination of zanthoxylum seeds. The significant enrichment KEGG pathways with the highest representation of the DEGs in profile 5 (water-treated seeds) and profile 6 (GAs-treated seeds) are shown in Table S6 and Table S7. Interestingly, metabolic pathways, phenylpropanoid biosynthesis, phenylalanine metabolism, flavonoid biosynthesis, plant hormone signal transduction appeared in both two tables. These pathways involved in substance metabolism, hormone regulation, antioxidant capacity and resistance regulation played important roles during the process of seed germination.

### Focused pathways and DEGs related to seed germination of zanthoxylum

We mainly focused on two pathways from above analysis, plant hormone signal transduction (ko04075 pathway) and phenylpropanoid biosynthesis (ko00940 pathway), which were involved in hormone regulation and resistance regulation. Every mapping KOID includes many homologous unigenes. According to the highest expression level of differentially expressed unigenes included in each KOID, three lowest levels of the filter thresholds of FPKM are selected: 100, 50, 25. By this means differentially expressed unigenes of three expression ranks are selected, which are helpful to investigate exhaustively the biological activity of pathways.

GAs function by means of removing the inhibitory DELLA proteins^34^. GA-GID-DELLA complex induces the association of DELLAs with the SLEEPY1 (SLY1)/GID2 F-box protein, which leads to DELLA proteins to be ubiquitinated by SLY1/GID2 and then to be degradated by the 26 S proteasome^35, 36^. The expression patterns of *DELLA* (*UNIGENE0058867*, *UNIGENE0058868*), *GID1* (*UNIGENE0057493*) and *SLY1* (*UNIGENE0026724*) were similar between the GAs-treated seeds and water-treated seeds, which indicated that the effect of the exogenous GAs on GA signaling was not significant (Figure S12).

9-cis-epoxycarotenoid dioxygenase (NCED) catalyzes oxidative cleavage of cis-epoxycarotenoid, which is the key step of the ABA biosynthesis^37^. Catabolic inactivation of ABA is mainly controlled by (+)-abscisic acid 8-hydroxylase (CYP707A)^38^. The expression of *NCED* (*UNIGENE0050269*) was lower, but that of *CYP707A* (*UNIGENE0077615*, *UNIGENE0077619*) was higher in W1/GA1/GA2 seeds (Figure S13), indicating a declining trend of ABA content. Our data showed the high level of expression of *PP2C* (*UNIGENE0043472*, *UNIGENE0054032*) and the low level of expression of *PYL*(*UNIGENE0043531*, *UNIGENE0043532*) in C0 seeds. It was reported that the exogenous ABA induced the up-regulated expression of the *ZmPP2C16* and the down-regulated expression of the *ZmPYL3* ^39^. We hypothesized the ABA content was rich in C0 seeds (Figure S14). In general, the exogenous GAs contributed to the mitigation of germination inhibition by ABA.

*TCH4* and *CYCD3* are vital response genes which play important roles in BR signaling. *TCH4* expression is strongly influenced by environmental and hormonal stimuli and the encoded protein acts on a major component of the plant cell wall. *TCH4* is hypothesized to function in cell wall modifications in response to environmental stress and during morphogenesis^40–42^. *CYCD3* is capable of stimulating supernumerary divisions^43^. The expressions of *TCH4* (*UNIGENE0068686*, *UNIGENE0044685*) and *CYCD3* (*UNIGENE0061155*) in GA2 seeds were the highest, which showed GA2 seeds were in the stage of growth and differentiation (Figure S15). The data showed BRs signaling functioned later than the first day of germination.

In JAs signaling, MYC2 recognizes the promoter of its target genes and regulates different branches of the JAs pathway^44^. MYC2 induces JAs-mediated responses such as wounding, root growth inhibition, JAs biosynthesis, oxidative stress adaptation and anthocyanin biosynthesis. JAZ proteins interact directly with MYC2 repressing its activity, which inhibits the JAs signaling pathway^45, 46^. The expressions of *MYC2* (*UNIGENE0075146*, *UNIGENE0057732*) and *JAZ* (*UNIGENE0035737*, *UNIGENE0044832*) in GA2 seeds were the highest, which promoted the anti-oxidative ability and defense ability against fungus infection during germination (Figure S16). The data also showed JAs pathway functioned later than the first day of germination.

Reactive oxygen species (ROS) play important roles as signaling molecules in plant development and growth and are produced as a result of aerobic metabolism, or in response to stresses^47, 48^. At the apoplast, ROS can be produced by respiratory burst oxidase homologue (RBOH) proteins, while many proteins are involved in ROS signaling, including FLS2, EFR, BAK1, BIK1, cyclic nucleotide gated channel (CNGF) and calcium-dependent protein kinase (CPK)^49–51^. The genes associated with ROS signaling including *CPK* (*UNIGENE0071915*, *UNIGENE0074165*), *RBOH* (*UNIGENE0060805*, *UNIGENE0072166*), *CML* (*UNIGENE0030801*, *UNIGENE0033873*), *CALM* (*UNIGENE0081076*, *UNIGENE0081077*), *CNGF* (*UNIGENE0072270*), *FLS2* (*UNIGENE0066275*), these genes in GA2 seeds showed the highest level of expression (Figure S17). The expression patterns implied that the GAs-treated seeds responsed to pathogenic fungi more efficiently in the second day of germination.

Phenylpropanoids involve the synthesis of lignin^26^. Lignin is an important part of the cell wall and plays an important role in the growth and development of plants and against pathogenic microorganisms. The genes associated with lignin biosynthesis including *phenylalanine ammonia-lyase* (*UNIGENE0075422*, *UNIGENE0075421*), *shikimate o−hydroxycinnamoyltransferase* (*UNIGENE0063533*, *UNIGENE0065728*), and *trans−cinnamate 4-monooxygenase* (*UNIGENE0053141*, *UNIGENE0039601*) were highly expressed in W1 seeds, which implied lignin biosynthesis was more active in W1 seeds (Figure S18).

According to the aforementioned genes, a cluster analysis of total differentially expressed unigenes was performed (Figure S19). C0 and W2 were clustered into 1 class, with a similar expression pattern. W1 and GA1 were clustered into 1 class. The expression pattern of GA2 is related to W1 and GA1. The clustering heatmap suggested that W2 and C0 were in the inhibited germination state, GA1 and W1 were in the initial preparation stage of germination during imbibition, and GA2 was in the differentiation stage of germination. The cluster analysis showed that the effects of the exogenous GAs promoting germination were more apparent in the second day of germination.

### Validation of differently expressed genes by qRT-PCR

**Figure 4:**
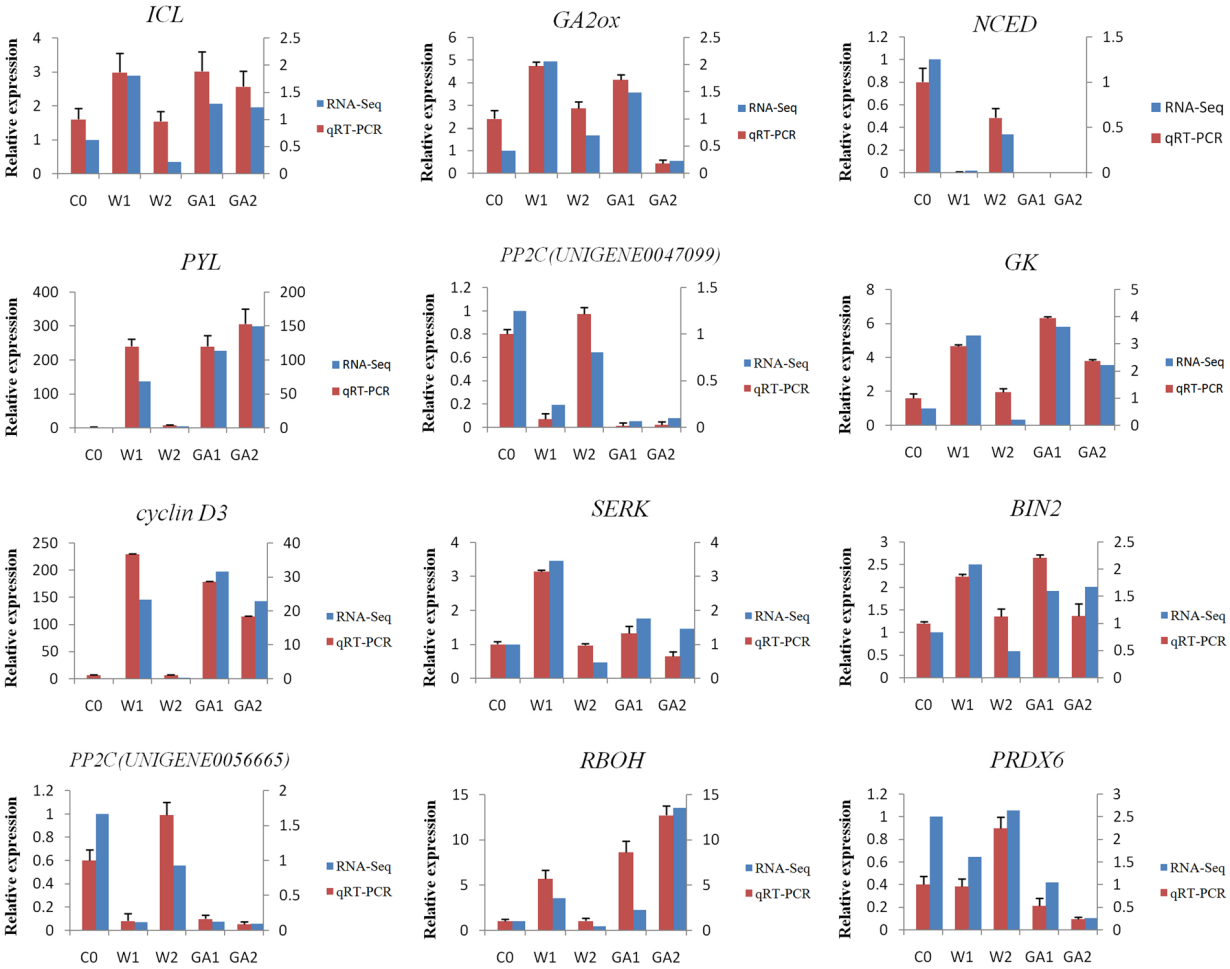
qRT-PCR validation of DEGs during germination of zanthoxylum seeds.

Twelve DEGs randomly selected were performed qRT-PCR to validate the RNA-seq data, They are isocitrate lyase(*ICL*, *UNIGENE0032088*), gibberellin 2-oxidase(*GA2ox*, *UNIGENE0045109*), 9-cis-epoxycarotenoid dioxygenase (*NCED*, *UNIGENE0050269*), abscisic acid receptor PYR/PYL (*PYL*, *UNIGENE0043532*), protein phosphatase 2 C (*PP2C*, *UNIGENE0047099*), glycerol kinase (*GK*, *UNIGENE0057496*), cyclin D3 (*UNIGENE0063918*), somatic embryogenesis receptor-like kinase (*SERK*, *UNIGENE0064764*), protein brassinosteroid insensitive 2 (*BIN2*, *UNIGENE0039997*), protein phosphatase 2 C (*PP2C*, *UNIGENE0056665*), respiratory burst oxidase (*RBOH*, *UNIGENE0072166*), 1-Cys peroxiredoxin (*PRDX6*, *UNIGENE0031506*). The results generated by qRT-PCR were compared to the data obtained by RNA-seq (Fig. 4). The reliability of the data obtained by RNA-seq was confirmed as the expression patterns of 9 DEGs by the two methods were similar.

## Disscusion

Comparing W1 and GA1 seeds during imbibition, there were similar expression patterns associated with a series of favorable germination events, such as *DELLA* down-regulation expression, the up-regulation expressions of the genes associated with the declining trend of ABA content and ROS signaling, and the genes related to lignin synthesis and antioxidant system were even higher expressed in W1 seeds. Although the expression patterns were similar during imbibition, the germination of the water-treated seeds was interrupted and the GAs-treated seeds continued to germinate in the second day of germination.

Interestingly, the genes related to RNA degradation were highly expressed in the water-treated seeds, but the genes related to splicing of development-related mRNAs were highly expressed in the GAs-treated seeds during imbibition. We thought that the active expression of *U6 snRNA−associated Sm−like protein LSm* marked a favorable germination tendency during imbibition. According to our data, we hypothesized the oxidative damages occurred in the water-treated seeds and increased RNA degradation, but the exogenous GAs promoted splicing of development-related mRNAs, indicating the GAs-treated seeds were in a favorable germination state during imbibition. Besides, the exogenous GAs up-regulated the expression of the genes related to ABA reduction, BR signaling, JA signaling and ROS signaling, promoting the germination of the GAs-treated seeds.

The production of high levels of ROS induces synthesis of antioxidant compounds and enhanced detoxifying activities of ROS such as SOD, peroxidases and other antioxidant like phenolics compounds^52^. More active lignin synthesis and antioxidant system mean more ROS product in the water-treated seeds during imbibition. H_2_O_2_ promotes seed dormancy breaking during imbibition^11^. However, ROS excess is able to cause a rapid decline of embryo viability^53^. We hypothesized H_2_O_2_ derived from β-oxidation promoted dormancy breaking, but H_2_O_2_ excess caused oxidative damages to the water-treated seeds during imbibitions, as a result the inhibitory factors of the germination including *NCED*, *PP2C*, *ABF*, *PRDX6* were up-regulated expression. Eventually, almost all of metabolic pathways were inactive transcription in the second day of the germination of the water-treated seed (Figure S19).

Generally, very little lipase activity is detected in imbibed seeds prior to germination^54^. Recently, it is also reported that the lipase activity is present in coffee seeds before imbibition and further induced by the germination process^55^. An early study reports that defects in peroxisomal β-oxidation dramatically reduce germination frequency in some cases^56^. TAG lipase, long−chain acyl−CoA synthetase and citrate synthase catalyze the initial steps of TAG breakdown, β-oxidation and citrate synthase, respectively. Impressively, TAG lipase catalyzes the first step of oil metabolisms. It is obvious that the down-regulated expressions of the above enzymes contribute to high efficient inhibitory of the oil metabolisms. As shown in Figure S7, the genes including *TAG lipase* were significantly down-regulated in the GAs-treated seeds, indicating that the exogenous GAs had inhibitory effects on the oil metabolisms during imbibition. On the country, the genes were active expressions in the water-treated seeds, indicating the active oil metabolisms and lipid peroxidation during imbibition. As a result, the germination of the water-treated seeds was inhibited due to oxidative damages brought from ROS. For the oil-rich seeds to germinate successfully, it was vital that the oil metabolisms were strictly regulated, and more specifically, a low activity of lipase was necessary during imbibition. Besides, we estimated that the proportion of GA/ABA of the zanthoxylum seeds was not conducive to germination, H_2_O_2_ promoted to break dormancy in the natural conditions.

Many reports about the relationship between GAs and sugar metabolism show GAs induced α-amylase synthesis and its activity to promote sugar metabolism during germination. Generally, the activation of the glyoxylation cycle of seeds oil utilization playes an importance positive role during germination. But few studies involved the relationship between GAs and oil metabolisms in imbibed seeds. Our data showed this relationship should not be underestimated during imbibition of oil-rich seeds. In this regard, the exogenous GAs played the vital inhibitory role in regulation of the oil metabolisms during imbibition. Due to the short of GAs in the seeds, it was expected that a gradual decomposition of the thick and oily shells could slowly increase the seeds permeability of water and air, retarding effectively the oil metabolisms in the initial stage of germination in the natural conditions. Thus, the suitable H_2_O_2_ content broke the dormancy while avoiding oxidative damages to imbibed seeds.

## Conclusion

We reported a comprehensive seed germination RNA-seq dataset of zanthoxylum generated by illumina paired-end sequencing technology. In total, 10,0982 unigenes from the seeds of zanthoxylum were assembled. The experimental data indicated the endogenous ABA of seeds was rich. The effects that the exogenous GAs promoted germination were apparent in the secong day of germination. Our results showed the key genes related to oil metabolisms such as *TAG lipase* and *long−chain acyl−CoA synthetase* were highly expressed in the water-treated seeds during imbibition. We hypothesized H_2_O_2_ excess derived from oil metabolisms caused oxidative damages and eventually up-regulated the expression of several inhibitory genes of germination such as *NCED*, *PP2C*, *ABF*, *PRDX6* during imbibition of the water-treated seeds. The exogenous GAs had inhibitory effects on the oil metabolisms to avoide oxidative damages to the GAs-treated seeds during imbibition. Because of the short of GAs in the seeds, the thick, dense and oily shell was indispensable to retarded effectively the aerobic metabolisms including the oil metabolisms and prevent oxidative damages in the initial stage of germination in the natural conditions.

## Methods

### Preparation of tissues for microscopic observation

The seeds of zanthoxylum were collected in Zhangjiajie, Hunan province, in December 2015. The newly collected seeds were cold-stratificated for three months. The seeds were soaked in 80% sulfuric acid for three minutes to remove shells easily. After the shell removal, some seeds were collected and labeled as C0, others were surface sterilized by washing in a 5% v/v solution of sodium hypochlorite (10–14% w/v available chlorine), then, soaked in gibberellins solution (1% w/v) for three and a half hours. Seeds were soaked in sterile water and used as control. Conditioning of the seeds took place on two sterile circles of filter paper, saturated with sterile double-distilled deionized water, in 9 cm perspex Petri dishes. Afterwards, the seeds were incubated in a controlled environment (day temperature 18 °C, night temperature 10 °C, day 16 hours, light intensity at bench level of 103 Wm^−2^). The water-treated seeds incubated for 1 day were labeled as W1, the water-treated seeds incubated for 2 days were labeled as W2, the GAs-treated seeds incubated for 1 day were labeled as GA1, the GAs-treated seeds incubated for 2 days were labeled as GA2. Seeds were fixed in FAA (50% alcohol: acetic acid: formaldehyde solution = 89: 6: 5) immediately after dissection, and stored at room temperature. Samples were washed in 50% alcohol, dehydrated using an ethyl alcohol series, cleared in xylene and embedded in paraffin wax. The specimens were sectioned to a thickness of 8 μm. Sections were stained with hematoxylin. Images were taken using an OLYMPUS BX-51 imaging system.

### Library construction and RNA-Seq

Total RNA was extracted using Trizol (Invitrogen, CA, USA), then incubated with RNase-free DNase I (Takara Bio, China) for 30 min at 37 °C to remove residual DNA. Quantity and quality of total RNA were assessed using NanoDrop 2000 (Thermo Scientific, Wilmington, DE, USA) and RNase free agarose gel electrophoresis. mRNA was purified using oligo-dT beads (Qiagen). Fragmentation was carried out by adding fragmentation buffer. First-strand cDNA was synthesized using random hexamer-primed reverse transcription. Second-strand cDNA was subsequently synthesized using DNA Polymerase I and RNase H. To select cDNA fragments at a preferential length about 150 bp to 200 bp, fragments were purified using a QIAquick PCR extraction kit. After washed with EB buffer, the gathered fragments were ligated to sequencing adapters. Then, PCR was performed to construct the final cDNA library. Finally, PCR products were purified and library quality was assessed on Agilent Bioanalyzer 2100 system. The seven cDNA library sequencing was carried out with Illiumina HiSeq. 2500 RNA-Seq (Illumina, San Diego, CA, USA) using the paired-end technology by Gene Denovo Company (Guangzhou, China).

### De novo assembly and functional annotation

Clean data was obtained by removing low-quality reads with adaptor or ambiguous bases (‘N’ < 10%), as well as all reads with more than 50% nucleotides that had Phred quality scores < 5. The high-quality reads were mapped to ribosome RNA (rRNA) to identify residual rRNA reads, the rRNA removed reads were clean reads. Then all clean reads of the seven libraries were jointly assembled into unigenes employed by Trinity software. Without a reference genome for zanthoxylum, a k-mer value cutoff of 25 was used after removing redundant nucleotide sequences by Tgicl (v2.1, http://sourceforge.net/projects/tgicl/files/tgicl%20v2.1/). The assembled unigenes were searched against the Nr (NCBI non-redundant protein sequences), Swissprot, COG (Clusters of Orthologous Groups of proteins), and KEGG (Kyoto Encyclopedia of Genes and Genomes) database using BlastX with an E-value < 10–5 to predict protein sequences, possible functional classifications and molecular pathways. GO annotation was performed with Blast2GO (https://www.blast2go.com/) and GO functional classification was carried out using WEGO (http://wego.genomics.org.cn/).

### Analysis of enrichment and dynamic expression profile of DEGs

The unique-match fragments were normalized to FPKM (fragments per kb per million reads) for gene expression analysis. DEGs between the two libraries (C0_vs_W1, W1_vs_W2, C0_vs_GA1, GA1_vs_GA2) were identified using DESeq R package(3.1.0). The p values were adjusted using Benjamini and Hochberg’s methods for controlling for the false discovery rate. In this experiment, DEGs between above libraries were restricted with p < 0.05 and the absolute value of log2 Ratio ≥ 2. Go enrichment analysis and KEGG pathway enrichment analysis of DEGs were performed using GOseq and KOBAS with p ≤ 0.05, respectively. Go enrichment analysis and KEGG pathway enrichment analysis based on Wallenius non-central hyper-geometric distribution model and hyper-geometric distribution model, respectively. DEGs were clustered by STEM^57^ with p-value ≤ 0.05, which indicated the clustered profiles were significant.

### Validation of RNA-Seq results by qRT-PCR

Twelve differentially expressed unigenes selected randomly were performed qRT-PCR: *ICL*, *GA2ox*, *NCED*, *PYL*, *PP2C*(two homologous unigenes), *GK*, *cyclin D3*, *SERK*, *BIN2*, *RBOH* and *PRDX6*. The extracted RNA of the seed samples (described above) were converted into cDNA using PrimeScriptTM One Step RT-PCR Kit Ver. 2 (Takara, China). The cDNA was 10 × diluted and used as templates for qPCR. Primers for qRT-PCR were designed using Primer Premier 5.0 software and synthesized by Sangon Biotech Co., Ltd (Shanghai). The zanthoxylum homologue actin was selected as reference. All the primers were shown in Table S8. qRT-PCR was performed on a Applied Biosystems 7500 Real-Time PCR System (ABI, USA) using a SYBR Green based PCR assay. Each reaction mixture was 10 μL containing 3 μL of diluted first-strand cDNAs and 125 nM of each primer, SYBR Green PCR Master Mix (TaKaRa, China) 5 μL. The two-step qRT-PCR was performed as follows: 95 °C for 30 s, followed by 40 cycles of 95 °C for 5 s, 57 °C for 30 s in 96-well optical reaction plates (ABI, USA). Each qRT-PCR analysis was performed in triplicate. Expression levels of the tested reference genes were determined by CT values and calculated by 2^−ΔΔCt^.

### Accession codes

SAMN07178644, SAMN07178645, SAMN07178646, SAMN07178647, SAMN07178648, SAMN07178649, SAMN07178650.

## Electronic supplementary material

Supplementary Information

## References

[1] Ma YZ, Wang P (2008). Evaluation of Threatened Degree and Sustainable Utilization Countermeasures of zanthoxylum Dissitum Resource(in Chinese). Nonwood Forest Research..

[2] Bewley JZ (1997). Seed germination and dormancy. The Plant Cell..

[3] Graham IA (2008). Seed storage oil mobilization. Annu Rev Plant Biol..

[4] Li-Beisson, Y. *et al*. Acyl-lipid metabolism. Arabidopis Book. 8:e0133 doi:10.1199/tab.0133 (2010). 10.1199/tab.0133 PMC3244904 22303259

[5] Russell L, Larner V, Kurup S, Bougourd S, Holdsworth M (2000). The Arabidopsis COMATOSE locus regulates germination potential. Development..

[6] Penfield S (2004). Reserve mobilization in the Arabidopsis endosperm fuels hypocotyl elongation in the dark, is independent of abscisic acid, and requires PHOSPHOENOLPYRUVATE CARBOXYKINASE1. Plant Cell..

[7] Pritchard SL, Charlton WL, Baker A, Graham IA (2002). Germination and storage reserve mobilization are regulated independently in Arabidopsis. Plant J..

[8] Corpas FJ, Barroso JB, del Río. LA (2001). Peroxisomes as a source of reactive oxygen species and nitric oxide signal molecules in plant cells. Trends Plant Sci..

[9] Lopez-Huertas E, Charlton WL, Johnson B, Graham IA, Baker A (2000). Stress induces peroxisome biogenesis genes. EMBO J..

[10] Ogawa K, Iwabuchi M (2001). A mechanism for promoting the germination of Zinnia elegans seeds by hydrogen peroxide. Plant and Cell Physiology..

[11] Bailly C, El-Maarouf-Bouteau H, Corbineau F (2008). From intracellular signaling networks to cell death: the dual role of reactive oxygen species in seed physiology. Comptes Rendu Biologies..

[12] Hu Y, Yu D (2014). BRASSINOSTEROID INSENSITIVE2 interacts with ABSCISIC ACID INSENSITIVE5 to mediate the antagonism of brassinosteroids to abscisic acid during seed germination in Arabidopsis. Plant Cell..

[13] White CN, Proebsting WM, Hedden P, Rivin CJ (2000). Gibberellins and seed development in maize. I. Evidence that gibberellin/abscisic acid balance governs germination versus maturation pathways. Plant Physiology..

[14] Yamauchi Y (2004). Activation of gibberellin biosynthesis and response pathways by low temperature during imbibition of Arabidopsis thaliana seeds. The Plant Cell..

[15] Ye N, Zhang J (2012). Antagonism between abscisic acid and gibberellins is partially mediated by ascorbic acid during seed germination in rice. Plant Signal Behav..

[16] Go’mez-Cadenas. A, Zentalla R, Walker-Simmons MK, Ho TH (2001). Gibberellin/abscisic acid antagonism in barley aleurone cells: site of action of the protein kinase PKABA1 in relation to gibberellin signaling molecules. The Plant Cell..

[17] Zentella R, Yamauchi D, Ho TH (2002). Molecular dissection of the gibberellin/abscisic acid signaling pathways by transiently expressed RNA interference in barley aleurone cells. The Plant Cell..

[18] Clouse SD (2011). Brassinosteroid signal transduction: from receptor kinase activation to transcriptional networks regulating plant development. Plant Cell..

[19] Wang ZY, Bai MY, Oh E, Zhu JY (2012). Brassinosteroid signaling network and regulation of photomorphogenesis. Annu Rev Genet..

[20] Xue LW (2009). Brassinosteroids counteract abscisic acid in germination and growth of Arabidopsis. Z Naturforsch C..

[21] Browse J, Howe GA (2008). New weapons and a rapid response against insect attack. Plant Physiol..

[22] Wasternack C (2007). Jasmonates: an update on biosynthesis, signal transduction and action in plant stress response, growth and development. Ann Bot (Lond)..

[23] Yang DL (2012). Plant hormone jasmonate prioritizes defense over growth by interfering with gibberellin signaling cascade. Proc Natl Acad Sci USA.

[24] Fernandez-Arbaizar A, Regalado JJ, Lorenzo O (2012). Isolation and characterization of novel mutant loci suppressing the ABA hypersensitivity of the Arabidopsis coronatine insensitive 1–16 (coi1-16) mutant during germination and seedling growth. Plant & Cell Physiology..

[25] Dixon RY (2002). The phenylpropanoid pathway and plant defence - a genomics perspective. Mol Plant Pathol..

[26] Harborne JB, Williamsm CA (2000). Advances in flavonoid research since 1992. Phytochemistry..

[27] Hasleks C (2003). Seed 1-cysteine peroxiredoxin antioxidants are not involved in dormancy, but contribute to inhibition of germination during stress. Plant Physiol..

[28] Cieplak MK (2011). miRNA-mediated deadenylation is orchestrated by GW182 through two conserved motifs that interact with CCR4-NOT. Nat. Struct. Mol. Biol..

[29] Sandler H, Kreth J, Timmers HT, Stoecklin G (2011). Not1 mediates recruitment of the deadenylase Caf1 to mRNAs targeted for degradation by tristetraprolin. Nucleic Acids Res..

[30] Ito K, Takahashi A, Morita M, Suzuki T, Yamamoto T (2011). The role of the CNOT1 subunit of the CCR4-NOT complex in mRNA deadenylation and cell viability. Protein. Cell..

[31] Fenger-Groen M, Fillman C, Norrild B, Lykke-Andersen J (2005). Multiple processing body factors and the ARE binding protein TTP activate mRNA decapping. Mol. Cell..

[32] Perea-Resa C, Hernandez-Verdeja T, Lopez-Cobollo R, del Mar Castellano M, Salinas J (2012). LSM proteins provide accurate splicing and decay of selected transcripts to ensure normal Arabidopsis development. Plant Cell..

[33] Golisz A, Sikorski PJ, Kruszka K, Kufel J (2013). Arabidopsis thaliana LSM proteins function in mRNA splicing and degradation. Nucleic Acids Res..

[34] Li QF, He JX (2013). Mechanisms of signaling crosstalk between brassinosteroids and gibberellins. EpubJul..

[35] McGinnis KM (2003). The Arabidopsis SLEEPY1 gene encodes a putative F-box subunit of an SCF E3 ubiquitin ligase. Plant Cell..

[36] Dill A, Thomas SG, Hu J, Steber CM, Sun TP (2004). The Arabidopsis F-box protein SLEEPY1 targets gibberellin signaling repressors for gibberellin-induced degradation. Plant Cell..

[37] Priya R, Siva R (2015). Analysis of phylogenetic and functional diverge in plant nine-cis epoxycarotenoid dioxygenase gene family. J Plant Res..

[38] Todoroki Y, Ueno K (2010). Development of specific inhibitors of CYP707A, a key enzyme in the catabolism of abscisic acid. Curr Med Chem..

[39] Wang YG (2014). Interaction between abscisic acid receptor PYL3 and protein phosphatase type 2C in response to ABA signaling in maize. Gene..

[40] Xu W, Campbell P, Vargheese AK, Braam J (1996). The Arabidopsis XETrelated gene family: environmental and hormonal regulation of expression. Plant J..

[41] Xu W (1995). Arabidopsis TCH4, regulated by hormones and the environment, encodes a xyloglucan endotransglycosylase. Plant Cell..

[42] Campbe P, Braam J (1999). Xyloglucan endotransglycosylases: diversity of genes, enzymes and potential wall-modifying functions. Trends Plant Sci..

[43] Riou-Khamlichi C, Huntley R, Jacqmard A, Murray JAH (1999). Cytokinin activation of Arabidopsis cell division through a D-type cyclin. Science..

[44] Chini A (2007). The JAZ family of repressors is the missing link in jasmonate signaling. Nature..

[45] Zhang Y, Turner JG (2008). Wound-induced endogenous jasmonates stunt plant growth by inhibiting mitosis. PLoS ONE..

[46] Kazan K, Manners JM (2008). Jasmonate signaling: toward an integrated view. Plant Physiol..

[47] Marino D, Dunand C, Puppo A, Pauly N (2012). A burst of plant NADPH oxidases. Trends Plant Sci..

[48] Suzuki N (2011). Respiratory burst oxidases: the engines of ROS signaling. Curr Opin Plant Biol..

[49] Wrzaczek M, Brosche M, Kangasjarvi J (2013). ROS signaling loops-production, perception, regulation. Curr Opin Plant Biol..

[50] Dubiella U (2013). Calcium-dependent protein kinase/NADPH oxidase activation circuit is required for rapid defense signal propagation. Proc Natl Acad Sci USA..

[51] Liu Y, He C (2016). Regulation of plant reactive oxygen species (ROS) in stress responses: learning from AtRBOHD. Plant Cell Rep..

[52] Espinosa F, Garrido I, Ortega A, Casimiro I, álvarez-Tinaut MC (2014). Redox activities and ROS, NO and phenylpropanoids production by axenically cultured intact olive seedling roots after interaction with a mycorrhizal or a pathogenic fungus. PLoS One..

[53] Pasquini S (2012). Seed storage in polyethylene bags of a recalcitrant species (Quercusilex): analysis of some bio-energetic and oxidative parameters. Acta Physiol.Plant..

[54] El-Kouhen K (2005). Identification and characterization of a triacylglycerol lipase in Arabidopsis homologous to mammalian acid lipases. FEBS Lett..

[55] Patui S (2014). Lipase activity and antioxidant capacity in coffee (Coffea arabica L.) seeds during germination. Plant Sci..

[56] Baker A, Graham IA, Holdsworth M, Smith SM, Theodoulou FL (2006). Chewing the fat: beta-oxidation in signaling and development. Trends Plant Sci..

[57] Ernst J, Bar-Joseph Z (2006). STEM: a tool for the analysis of short time series gene expression data. BMC Bioinformatics..

