# Supplementary material for: Transcriptome Analysis of the Effects of Shell Removal and Exogenous Gibberellin on Germination of Zanthoxylum Seeds
Source: Sci Rep. 2017 Aug 17;7:8521. doi: 10.1038/s41598-017-07424-0 (PMC5561108; doi:10.1038/s41598-017-07424-0)
Supplement: Supplementary file 1 — Supplementary Information [file 41598_2017_7424_MOESM1_ESM.pdf]

# Transcriptome Analysis of the Effects of Shell Removal and Exogenous Gibberellin on Germination of Zanthoxylum Seeds

Jikang Sun[1], Ping WANG[2*], Tao Zhou[1], Jian Rong[1], Hao Jia[1], Zhiming Liu[3]

1 College of Life Science and Technology, Central South University of Forestry and Technology, Changsha, Hunan, China

2 College of Environmental Science and Engineering, Central South University of Forestry and Technology, Changsha, Hunan, China

3 Department of Biology, Eastern New Mexico University, Portales, NM88130, USA.

## E-mail address for all authors:

Sun JK: 1825856598@qq.com
Wang P: wangping@csuft.edu.cn, *Corresponding author
Zhou T: 1505726228@qq.com
Rong J: 1152719851@qq.com
Jia H: 1146670035@qq.com
Liu ZM: Zhiming.Liu@enmu.edu

## Supplementary Tables and Figures

### Table S1. Summary of the assembly

| Genes Num | GC percentage | N50 | Max length | Min length | Average length | Total assembled bases |
|---|---|---|---|---|---|---|
| 100982 | 41.99% | 944 | 18760 | 201 | 636.41 | 64266356 |

N50: the length L where 50% of all nucleotides in the assembly are contained in unigenes of size ≥L.

### Table S2. the top 20 annotation KEGG pathways

| Pathway ID | Pathway define | Count |
|---|---|---|
| ko01100 | Metabolic pathways | 5020 |
| ko01110 | Biosynthesis of secondary metabolites | 2617 |
| ko03010 | Ribosome | 1930 |
| ko04141 | Protein processing in endoplasmic reticulum | 833 |
| ko00190 | Oxidative phosphorylation | 751 |
| ko03013 | RNA transport | 693 |
| ko03040 | Spliceosome | 585 |
| ko00010 | Glycolysis / Gluconeogenesis | 539 |
| ko00230 | Purine metabolism | 503 |
| ko04626 | Plant-pathogen interaction | 472 |
| ko04075 | Plant hormone signal transduction | 459 |
| ko03018 | RNA degradation | 451 |
| ko04120 | Ubiquitin mediated proteolysis | 425 |
| ko03015 | mRNA surveillance pathway | 387 |
| ko00620 | Pyruvate metabolism | 383 |
| ko04145 | Phagosome | 372 |
| ko00240 | Pyrimidine metabolism | 352 |
| ko00020 | Citrate cycle (TCA cycle) | 347 |
| ko04144 | Endocytosis | 339 |

**Table S3. GO enrichment analysis of differentially expressed genes**

| | GO ID | Description | Unigene | Pvalue | Qvalue |
|---|---|---|---|---|---|
| **molecular function** | GO:0005198 | structural molecule activity | 1092 | 4.87E-42 | 3.40E-39 |
| | GO:0016788 | hydrolase activity, acting on ester | 834 | 0.000656 | 0.038231 |
| | GO:0001071 | nucleic acid binding transcription | 708 | 2.24E-07 | 5.63E-05 |
| | GO:0003723 | RNA binding | 648 | 2.41E-07 | 5.63E-05 |
| | GO:0003677 | DNA binding | 540 | 1.87E-06 | 0.000327 |
| | GO:0016628 | oxidoreductase activity, acting on the | 303 | 0.000272 | 0.021549 |
| | GO:0008194 | UDP-glycosyltransferase activity | 224 | 6.06E-05 | 0.00706 |
| **biological process** | GO:0065007 | biological regulation | 4456 | 8.78E-07 | 0.000135 |
| | GO:0050789 | regulation of biological process | 4136 | 7.33E-05 | 0.004567 |
| | GO:0050896 | response to stimulus | 3791 | 4.38E-08 | 2.63E-05 |
| | GO:0009058 | biosynthetic process | 3517 | 0.000914 | 0.029932 |
| | GO:1901576 | organic substance biosynthetic process | 3277 | 0.001223 | 0.034964 |
| | GO:0044249 | cellular biosynthetic process | 3243 | 0.000742 | 0.026217 |
| | GO:0019222 | regulation of metabolic process | 2681 | 9.42E-05 | 0.005658 |
| | GO:0006810 | transport | 2622 | 0.000356 | 0.016049 |
| | GO:0060255 | regulation of macromolecule | 2311 | 0.000925 | 0.029932 |
| | GO:0010467 | gene expression | 2065 | 3.06E-10 | 5.51E-07 |
| | GO:1902578 | single-organism localization | 1956 | 1.94E-07 | 5.00E-05 |
| | GO:0006950 | response to stress | 1940 | 1.13E-05 | 0.001134 |
| | GO:0044765 | single-organism transport | 1938 | 1.59E-07 | 4.77E-05 |
| | GO:0042221 | response to chemical | 1730 | 1.49E-07 | 4.77E-05 |
| | GO:0051716 | cellular response to stimulus | 1681 | 0.001179 | 0.034822 |
| | GO:0007154 | cell communication | 1260 | 0.000349 | 0.016049 |
| | GO:0071702 | organic substance transport | 1212 | 0.002052 | 0.049281 |
| | GO:0023052 | signaling | 1172 | 6.68E-05 | 0.004567 |
| | GO:0044700 | single organism signaling | 1172 | 6.68E-05 | 0.004567 |
| | GO:0007165 | signal transduction | 1164 | 7.35E-05 | 0.004567 |
| **cellular component** | GO:0005623 | cell | 9882 | 0.000568 | 0.005359 |
| | GO:0044464 | cell part | 9881 | 0.000599 | 0.005451 |
| | GO:0044444 | cytoplasmic part | 3958 | 0.001319 | 0.010836 |
| | GO:0044422 | organelle part | 3011 | 3.88E-17 | 3.30E-15 |
| | GO:0032991 | macromolecular complex | 2834 | 8.53E-07 | 1.36E-05 |
| | GO:0044446 | intracellular organelle part | 2491 | 1.64E-12 | 6.95E-11 |
| | GO:0009536 | plastid | 2020 | 7.81E-11 | 1.81E-09 |
| | GO:0030529 | intracellular ribonucleoprotein | 1475 | 5.90E-34 | 7.52E-32 |
| | GO:1990904 | ribonucleoprotein complex | 1475 | 5.90E-34 | 7.52E-32 |
| | GO:0031090 | organelle membrane | 1341 | 9.42E-16 | 6.00E-14 |
| | GO:0044435 | plastid part | 987 | 1.33E-11 | 3.76E-10 |
| | GO:0031967 | organelle envelope | 781 | 7.19E-12 | 2.29E-10 |

| | | | | |
|---|---|---|---|---|
| GO:0031975 | envelope | 781 | 7.19E-12 | 2.29E-10 |
| GO:0009526 | plastid envelope | 496 | 4.96E-09 | 9.71E-08 |
| GO:0005840 | ribosome | 490 | 1.25E-13 | 6.38E-12 |
| GO:0009532 | plastid stroma | 475 | 9.06E-06 | 0.000128 |
| GO:0071944 | cell periphery | 428 | 9.04E-05 | 0.001002 |
| GO:0044391 | ribosomal subunit | 375 | 6.24E-11 | 1.59E-09 |
| GO:0030312 | external encapsulating structure | 371 | 3.98E-06 | 5.96E-05 |
| GO:0031984 | organelle subcompartment | 325 | 1.52E-09 | 3.22E-08 |

The top 20 significant enrichment GO terms (Qvalue<0.05) of DEGs was listed in each of three main categories: biological process, cellular component and molecular function.

**Table S4. KEGG enrichment pathways analysis of up-regulated unigenes and down-regulated unigenes during imbibitions, respectively.**

| | Pathway ID | Pathway define | Tendency unigenes | Pathway unigenes | Qvalue |
|---|---|---|---|---|---|
| C0-VS-W1 (up-regulated) | ko03010 | Ribosome | 555 | 1930 | 2.54E-36 |
| | ko01110 | Biosynthesis of secondary metabolites | 628 | 2617 | 3.78E-18 |
| | ko01100 | Metabolic pathways | 1085 | 5020 | 4.50E-17 |
| | ko00940 | Phenylpropanoid biosynthesis | 84 | 225 | 2.68E-11 |
| | ko00941 | Flavonoid biosynthesis | 29 | 52 | 1.23E-08 |
| | ko00945 | Stilbenoid, diarylheptanoid and gingerol | 23 | 49 | 3.73E-05 |
| | ko00020 | Citrate cycle (TCA cycle) | 93 | 347 | 1.45E-04 |
| | ko00010 | Glycolysis / Gluconeogenesis | 133 | 539 | 1.87E-04 |
| | ko00710 | Carbon fixation in photosynthetic organisms | 76 | 277 | 3.05E-04 |
| | ko00511 | Other glycan degradation | 15 | 31 | 8.98E-04 |
| | ko04626 | Plant-pathogen interaction | 115 | 472 | 9.03E-04 |
| | ko00360 | Phenylalanine metabolism | 47 | 161 | 1.65E-03 |
| | ko00400 | Phenylalanine, tyrosine and tryptophan | 37 | 119 | 1.84E-03 |
| | ko00592 | alpha-Linolenic acid metabolism | 36 | 120 | 4.43E-03 |
| | ko00053 | Ascorbate and aldarate metabolism | 34 | 116 | 9.14E-03 |
| | ko00071 | Fatty acid metabolism | 58 | 227 | 1.03E-02 |
| | ko00270 | Cysteine and methionine metabolism | 70 | 288 | 1.40E-02 |
| | ko00900 | Terpenoid backbone biosynthesis | 37 | 135 | 1.78E-02 |
| | ko00531 | Glycosaminoglycan degradation | 11 | 27 | 2.40E-02 |
| | ko04075 | Plant hormone signal transduction | 102 | 459 | 3.02E-02 |
| | ko00604 | Glycosphingolipid biosynthesis - ganglio series | 6 | 11 | 3.34E-02 |
| | ko00500 | Starch and sucrose metabolism | 76 | 331 | 3.41E-02 |
| | ko00620 | Pyruvate metabolism | 86 | 383 | 3.71E-02 |
| C0-VS-W1 (down-regulate | ko04075 | Plant hormone signal transduction | 48 | 459 | 0.000164 |
| | ko04712 | Circadian rhythm - plant | 16 | 85 | 0.000243 |
| | ko00860 | Porphyrin and chlorophyll metabolism | 17 | 126 | 0.007008 |
| | ko03450 | Non-homologous end-joining | 4 | 11 | 0.03268 |
| | ko00196 | Photosynthesis - antenna proteins | 6 | 28 | 0.040276 |

| | Pathway ID | Pathway define | Tendency unigenes | Pathway unigenes | Qvalue |
|---|---|---|---|---|---|
| C0-VS-GA1 (up-regulated) | ko03010 | Ribosome | 588 | 1930 | 6.06E-54 |
| | ko00940 | Phenylpropanoid biosynthesis | 78 | 225 | 2.98E-09 |
| | ko00941 | Flavonoid biosynthesis | 29 | 52 | 7.59E-09 |
| | ko00195 | Photosynthesis | 39 | 98 | 1.50E-06 |
| | ko00945 | Stilbenoid, diarylheptanoid and gingerol | 24 | 49 | 4.61E-06 |
| | ko04626 | Plant-pathogen interaction | 108 | 472 | 6.87E-03 |
| | ko00360 | Phenylalanine metabolism | 43 | 161 | 1.67E-02 |
| C0-VS-GA1 (down-regulated) | ko04075 | Plant hormone signal transduction | 107 | 459 | 2.84E-10 |
| | ko03018 | RNA degradation | 100 | 451 | 1.51E-08 |
| | ko04144 | Endocytosis | 79 | 339 | 7.10E-08 |
| | ko04120 | Ubiquitin mediated proteolysis | 84 | 425 | 3.84E-05 |
| | ko04141 | Protein processing in endoplasmic reticulum | 139 | 833 | 3.27E-04 |
| | ko04712 | Circadian rhythm - plant | 22 | 85 | 5.16E-03 |
| | ko03015 | mRNA surveillance pathway | 68 | 387 | 8.31E-03 |
| | ko00565 | Ether lipid metabolism | 15 | 51 | 8.31E-03 |
| | ko00860 | Porphyrin and chlorophyll metabolism | 28 | 126 | 8.56E-03 |
| | ko00514 | Other types of O-glycan biosynthesis | 4 | 6 | 2.66E-02 |
| | ko00561 | Glycerolipid metabolism | 28 | 137 | 2.66E-02 |
| | ko00564 | Glycerophospholipid metabolism | 33 | 172 | 3.06E-02 |
| | ko04070 | Phosphatidylinositol signaling system | 26 | 127 | 3.06E-02 |
| | ko00590 | Arachidonic acid metabolism | 11 | 39 | 3.56E-02 |
| | ko03040 | Spliceosome | 90 | 585 | 3.99E-02 |
| | ko03450 | Non-homologous end-joining | 5 | 11 | 4.04E-02 |

| | Pathway ID | Pathway define | Tendency unigenes | Pathway unigenes | Qvalue |
|---|---|---|---|---|---|
| W1-VS-GA1 (up-regulated) | ko03010 | Ribosome | 263 | 1930 | 4.81E-58 |
| | ko00195 | Photosynthesis | 22 | 98 | 4.35E-08 |
| W1-VS-GA1 (down-regulated) | ko03018 | RNA degradation | 99 | 451 | 3.56E-07 |
| | ko04144 | Endocytosis | 78 | 339 | 9.98E-07 |
| | ko03010 | Ribosome | 308 | 1930 | 6.78E-06 |
| | ko04075 | Plant hormone signal transduction | 94 | 459 | 7.18E-06 |
| | ko04141 | Protein processing in endoplasmic reticulum | 146 | 833 | 7.28E-05 |
| | ko03013 | RNA transport | 120 | 693 | 7.88E-04 |
| | ko04626 | Plant-pathogen interaction | 85 | 472 | 2.29E-03 |
| | ko00591 | Linoleic acid metabolism | 13 | 39 | 6.89E-03 |
| | ko00592 | alpha-Linolenic acid metabolism | 27 | 120 | 1.41E-02 |
| | ko03015 | mRNA surveillance pathway | 66 | 387 | 3.28E-02 |
| | ko00450 | Selenocompound metabolism | 22 | 98 | 3.28E-02 |

The significant enrichment KEGG pathway (Qvalue<0.05).

## Table S5. GO enrichment analysis of profile 5 (water-treated seeds) and profile 6 (GAs-treated seeds)

| | profile 5 (water-treated seeds) | | profile 6 (GAs-treated seeds) | |
|---|---|---|---|---|
| | **GO term** | **Qvalue** | **GO term** | **Qvalue** |
| **biological process** | carboxylic acid metabolic | 1.43E-19 | cellular metabolic compound salvage | 1.95E-13 |
| | oxoacid metabolic | 4.24E-19 | single-organism metabolic | 5.55E-11 |
| | organic acid metabolic | 4.64E-19 | proteasome assembly | 3.41E-07 |
| | small molecule metabolic | 2.94E-18 | response to organic substance | 7.81E-07 |
| | single-organism metabolic | 5.50E-18 | carboxylic acid metabolic | 6.56E-06 |
| | monocarboxylic acid metabolic | 1.51E-13 | sulfur amino acid biosynthetic | 6.78E-06 |
| | pyruvate metabolic | 1.95E-12 | pyruvate metabolic | 9.85E-06 |
| | cellular amino acid metabolic | 3.98E-12 | oxoacid metabolic | 1.07E-05 |
| | sulfur compound metabolic | 1.22E-11 | organic acid metabolic | 1.12E-05 |
| | cellular metabolic compound salvage | 3.50E-11 | single-organism metabolic | 1.14E-05 |
| **molecular function** | structural molecule activity | 1.99E-18 | structural molecule activity | 3.97E-10 |
| | oxidoreductase activity | 7.37E-06 | oxidoreductase activity, acting on paired donors, with incorporation or reduction of molecular oxygen | 5.08E-05 |
| | catalytic activity | 1.45E-05 | oligosaccharyl transferase activity | 6.92E-05 |
| | oxidoreductase activity, acting on CH-OH group of donors | 3.30E-05 | oxidoreductase activity, acting on CH-OH group of donors | 0.00025 |
| | hydrolase activity, hydrolyzing O-glycosyl compounds | 4.20E-05 | oxidoreductase activity | 0.00046 |
| | oxidoreductase activity, acting on the CH-OH group of donors, NAD or NADP as acceptor | 6.73E-05 | oxidoreductase activity, acting on the CH-OH group of donors, NAD or NADP as acceptor | 0.00069 |
| | hydrolase activity, acting on glycosyl bonds | 0.00018 | tetrapyrrole binding | 0.0011 |
| | intramolecular oxidoreductase activity | 0.00039 | O-methyltransferase activity | 0.00111 |
| | O-methyltransferase activity | 0.0004 | hydrolase activity, acting on glycosyl bonds | 0.00304 |
| | oxidoreductase activity, acting on paired donors, with incorporation or reduction of molecular oxygen | 0.00049 | fatty acid synthase activity | 0.00622 |
| **cellular component** | membrane part | 7.47E-12 | mitochondrial membrane part | 5.97E-12 |
| | organelle part | 9.88E-12 | mitochondrial membrane | 1.08E-11 |
| | organelle membrane | 4.71E-11 | mitochondrial envelope | 3.45E-11 |
| | mitochondrial part | 3.30E-10 | membrane part | 1.11E-10 |
| | organelle envelope | 5.44E-10 | organelle envelope | 3.07E-10 |
| | envelope | 5.44E-10 | envelope | 3.07E-10 |
| | intrinsic component of membrane | 9.84E-10 | mitochondrial part | 6.23E-10 |
| | mitochondrial membrane part | 1.56E-09 | external encapsulating structure | 5.51E-09 |
| | mitochondrial membrane | 2.60E-09 | cell wall | 1.01E-07 |
| | cell wall | 7.60E-09 | organelle part | 9.13E-07 |

The top 10 significant enrichment GO terms (Qvalue<0.05) of profile 5 (water-treated seeds) and profile 6

(GAs-treated seeds) were listed in each of three main categories: biological process, cellular component and molecular function.

**Table S6. KEGG enrichment pathways of DEGs in profile 5(water-treated seeds)**

| Pathway ID | Pathway define | unigenes | Pvalue | Qvalue |
|---|---|---|---|---|
| ko03010 | Ribosome | 423 | 1.96E-19 | 2.32E-17 |
| ko01100 | Metabolic pathways | 872 | 5.85E-10 | 2.95E-08 |
| ko01110 | Biosynthesis of secondary metabolites | 490 | 7.49E-10 | 2.95E-08 |
| ko00940 | Phenylpropanoid biosynthesis | 66 | 1.22E-08 | 3.61E-07 |
| ko00941 | Flavonoid biosynthesis | 23 | 2.97E-07 | 7.02E-06 |
| ko00400 | Phenylalanine, tyrosine and tryptophan biosynthesis | 35 | 2.87E-05 | 5.30E-04 |
| ko00945 | Stilbenoid, diarylheptanoid and gingerol biosynthesis | 19 | 3.14E-05 | 5.30E-04 |
| ko00360 | Phenylalanine metabolism | 43 | 5.05E-05 | 7.45E-04 |
| ko04626 | Plant-pathogen interaction | 98 | 0.000199 | 2.61E-03 |
| ko00710 | Carbon fixation in photosynthetic organisms | 60 | 0.00111 | 1.31E-02 |
| ko00900 | Terpenoid backbone biosynthesis | 32 | 0.00363 | 3.89E-02 |
| ko04075 | Plant hormone signal transduction | 88 | 0.004795 | 4.71E-02 |

The significant enrichment KEGG pathway (Qvalue<0.05).

**Table S7. KEGG enrichment pathways of DEGs in profile 6 (GAs-treated seeds)**

| Pathway ID | Pathway define | unigenes | Pvalue | Qvalue |
|---|---|---|---|---|
| ko00940 | Phenylpropanoid biosynthesis | 64 | 5.82E-14 | 6.87E-12 |
| ko03010 | Ribosome | 294 | 1.17E-11 | 6.89E-10 |
| ko01100 | Metabolic pathways | 643 | 1.70E-09 | 6.70E-08 |
| ko00941 | Flavonoid biosynthesis | 20 | 1.19E-07 | 3.52E-06 |
| ko00945 | Stilbenoid, diarylheptanoid and gingerol biosynthesis | 19 | 2.12E-07 | 5.00E-06 |
| ko01110 | Biosynthesis of secondary metabolites | 350 | 5.36E-07 | 1.05E-05 |
| ko00360 | Phenylalanine metabolism | 34 | 5.93E-05 | 1.00E-03 |
| ko04075 | Plant hormone signal transduction | 75 | 8.04E-05 | 1.19E-03 |
| ko00500 | Starch and sucrose metabolism | 57 | 0.000138 | 1.80E-03 |
| ko00510 | N-Glycan biosynthesis | 24 | 0.001052 | 1.24E-02 |
| ko00195 | Photosynthesis | 21 | 0.00118 | 1.27E-02 |
| ko00511 | Other glycan degradation | 9 | 0.00371 | 3.65E-02 |

The significant enrichment KEGG pathway (Qvalue<0.05).

## Table S8. Primer sequences of the DEGs for qRT-PCR

| Gene | Forward primer(5'-3') | Reverse primer(5'-3') | Length(bp) |
|---|---|---|---|
| *ICL* | CCAAAGACTATGCCAGAAGAGG | AGTTAGCGCCGGACCATT | 111 |
| *GA2ox* | ACCAAACCTCAGCTCACTTCC | CTGCCGTTATGTACTCATCCAC | 94 |
| *NCED* | TCGTGCAAGAAGCTGAATACG | AGCCGAGCCAGACCCAAG | 82 |
| *PYL* | CGCCACCGTATGGTCTGTC | CACTTCGCCGTCTCCGTTA | 97 |
| *PP2C* | CTTTCACGACAAGGACGGAT | CTCCCCAACTTCTTCATTCG | 89 |
| *GK* | GCAAGCGAGATCGAGGAAT | ATCACGCCACCAAGGAGC | 99 |
| cyclin D3 | GAGGCTGTTGATTGGGTGTT | CTTGGGTCTCATCCACTTTAGC | 187 |
| *SERK* | GGGTAGAGGTGGATTTGGTAAG | TGCAAAAGCCATGTAGACGA | 176 |
| *BIN2* | ATGAACTTTTCCTCAACTTGGTTA | GAGTGGCATCCTCTGGTTTG | 95 |
| *PP2C* | GGAGTGCGATGCTGTTGG | TCCTGCTTCTTGGATACGGT | 178 |
| *RBOH* | TAAAGTAGGAGACAGTCGAAACCTG | GCCATTACTCCAATCCAAAGTG | 162 |
| *PRDX6* | TGCAGAAGGCAATGAAGCA | TCTTGGATGGAAGGTCAACAGT | 144 |

Gene abbreviations: isocitrate lyase(*ICL, Unigene0032088*), gibberellin 2-oxidase(*GA2ox, Unigene0045109*), 9-cis-epoxycarotenoid dioxygenase (*NCED, UNIGENE0050269*), abscisic acid receptor PYR/PYL (*PYL, UNIGENE0043532*), protein phosphatase 2C (*PP2C, UNIGENE0047099*), glycerol kinase(*GK, UNIGENE0057496*), cyclin D3 (*UNIGENE0063918*), somatic embryogenesis receptor-like kinase (*SERK, UNIGENE0064764*), protein brassinosteroid insensitive 2 (*BIN2, UNIGENE0039997*), protein phosphatase 2C (*PP2C, UNIGENE0056665*), respiratory burst oxidase (*RBOH, UNIGENE0072166*), 1-Cys peroxiredoxin (*PRDX6, UNIGENE0031506*).

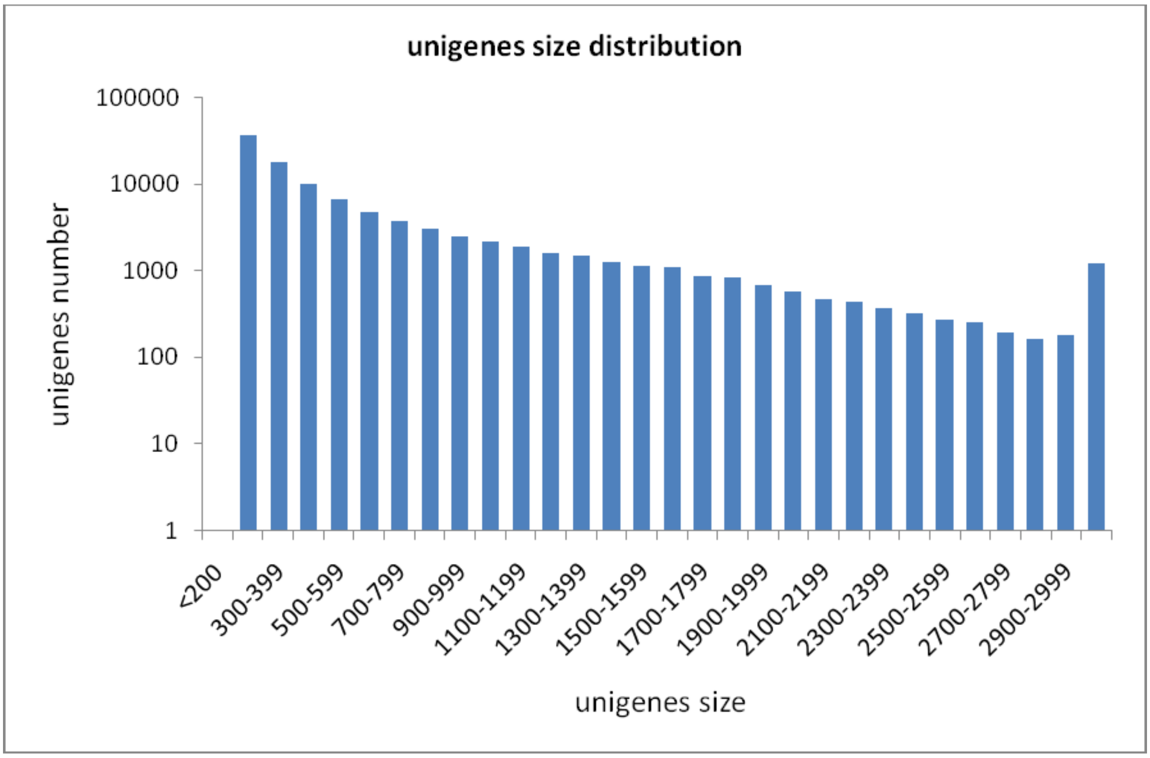


**Figure S1. The size distribution of the unigenes**

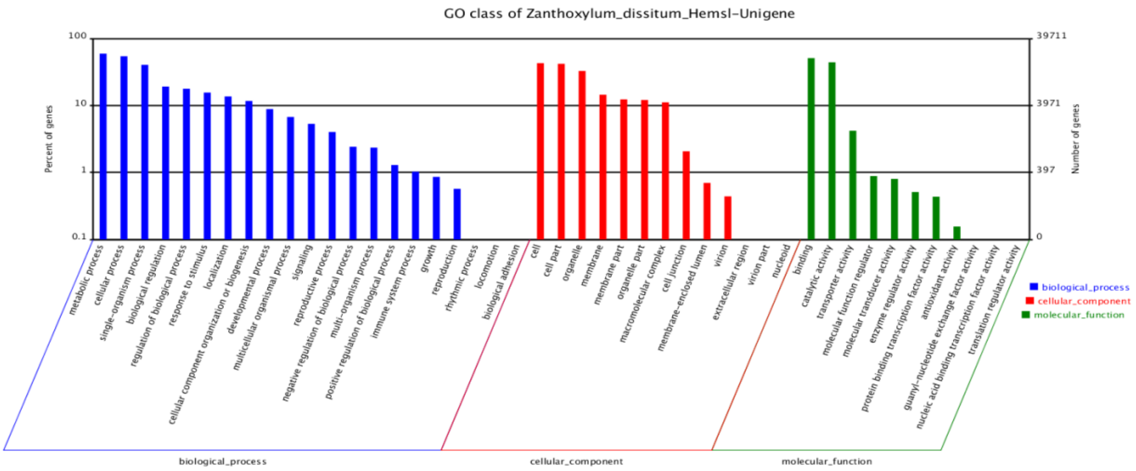


**Figure S2. Gene ontology classification of the unigenes from seeds of zanthoxylum.** The results are summarized in three main categories: biological process, cellular component and molecular function. In total, 57150 unigenes with BLAST matches to Nr database were assigned to gene ontology groups. Under the biological process category, the major GO terms were metabolic process (23701 unigenes), cellular process (21659 unigenes), single-organism process (16124 unigenes). Within the cellular component category, a significant percentage of genes were clustered into cell (17090 unigenes), cell part (16711 unigenes), organelle (13067 unigenes). In the molecular function category, most genes were assigned to binding (20319 unigenes), and catalytic activity (17606 unigenes).

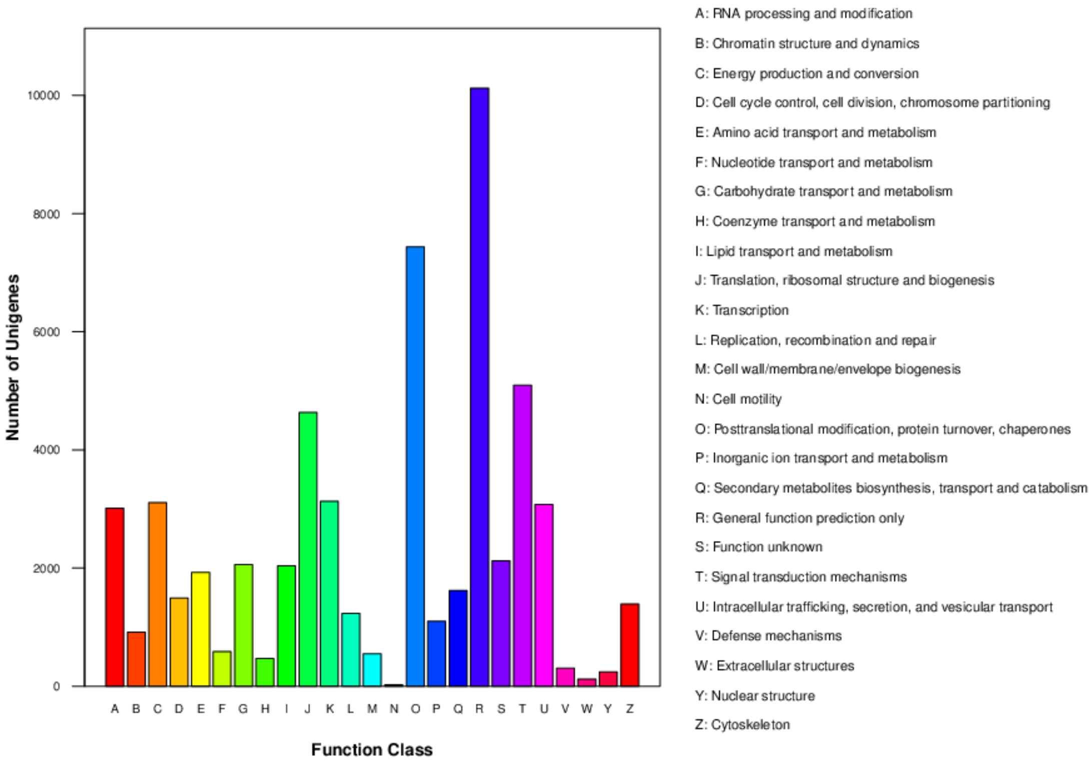


**Figure S3. KOG function classifications of unigenes from seeds of zanthoxylum.** Among them, the cluster for general function prediction only represented the largest group (10120 unigenes, 28.17%), followed by posttranslational modification, protein turnover, chaperones (7437 unigenes, 20.7%), signal transduction mechanisms (5094 unigenes, 14.18%), translation, ribosomal structure and biogenesis (4634 unigenes, 12.9%), transcription (3130 unigenes, 8.71%), energy production and conversion (3108 unigenes, 8.65%), intracellular trafficking, secretion, and vesicular transport (3076 unigenes, 8.56%), RNA processing and modification (3011 unigenes, 8.38%), function unknown (2124 unigenes, 5.91%), carbohydrate transport and metabolism (2058 unigenes, 5.73%), lipid transport and metabolism (2037 unigenes, 5.67%), amino acid transport and metabolism (1928 unigenes, 5.36%), secondary metabolites biosynthesis, transport and catabolism (1620 unigenes, 4.51%).

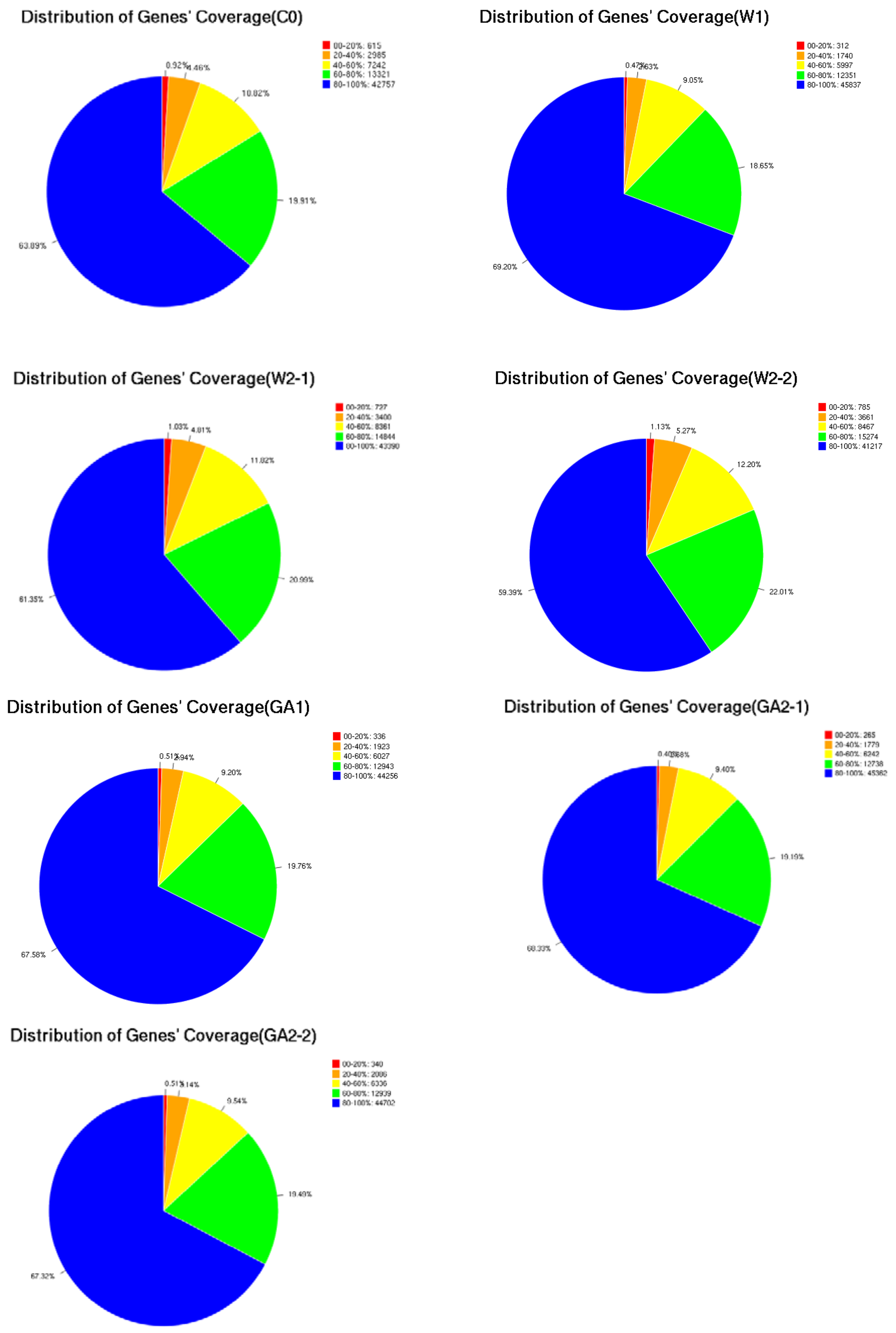


**Figure S4. The distribution of unigenes' coverage in each DGE library.** The different colors represent the proportion of unigenes with a certain coverage range to the all unigenes in each DGE library.

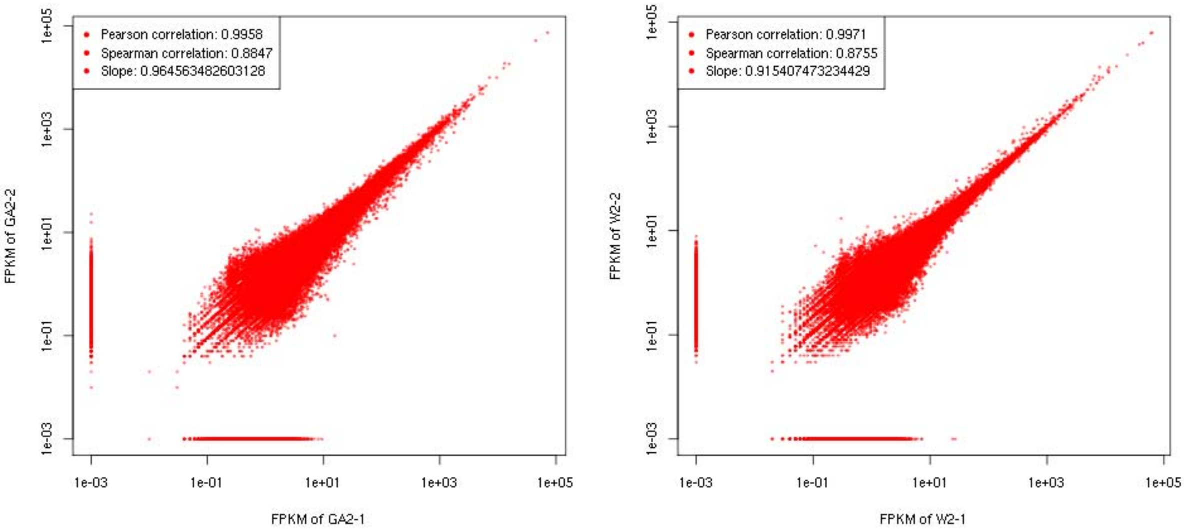


**Figure S5. Correlation analysis of unigenes expression (FPKM) between the biological replicates.** The left is the biological replicates of GAs-treated seeds germinating for 2 days with pearson correlation 0.9958, spearman correlation 0.8847 (GA2-1 vs GA2-2). The right is the biological replicates of water-treated seeds germinating for 2 days with pearson correlation 0.9971, spearman correlation 0.8755 (W2-1 vs W2-2).

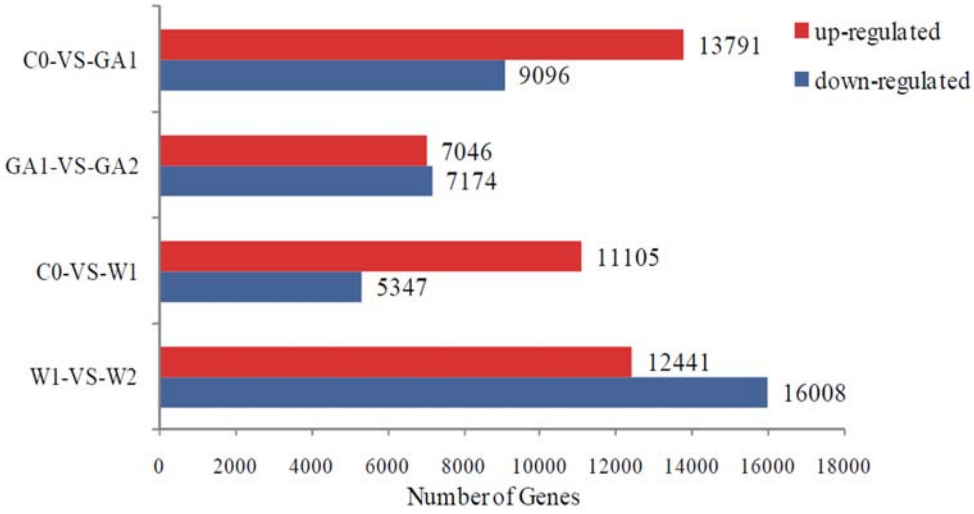


**Figure S6. Numbers of differentially expressed unigenes comparing different germination stages of the zanthoxylum seeds.** 22,887 between stages C0 and GA1 (60.25% up- and 39.74% down-regulated in stage GA1), 14,214 between stages GA1 and GA2 (49.53% up- and 50.47% down-regulated in stage GA2), 16,452 between stages C0 and W1 (67.5% up- and 32.5% down-regulated in stage W1), 28,449 between stages W1 and W2 (43.73% up- and 56.27% down-regulated in stage W2)

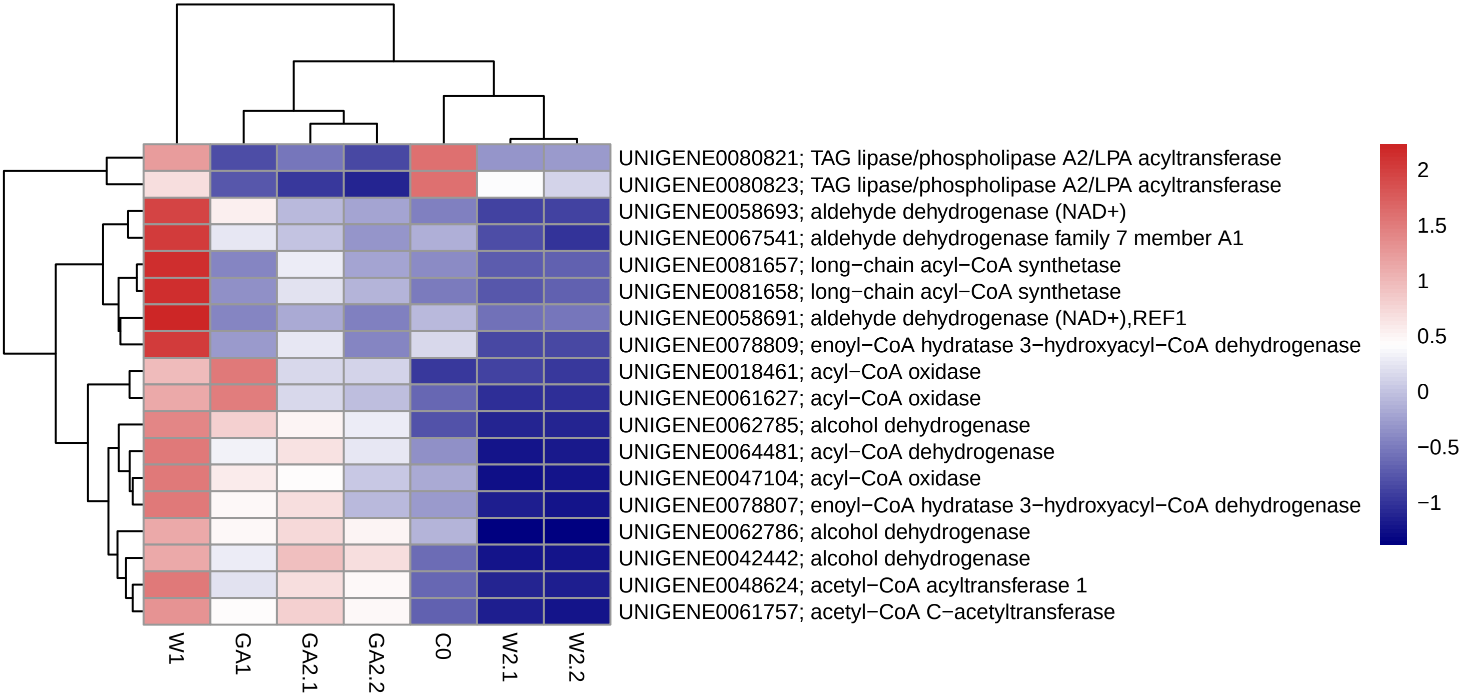


Figure S7. Heatmap diagram of expression levels for focused DEGs annotated in oil metabolism. The annotated unigenes are indicated at the side of each step. The sample names are showed at the bottom: C0 the seeds cold-stratificated for three months; W1 water-treated seeds germinating for 1 days; GA1 GAs-treated seeds germinating for 1 days; W2.1,W2.2 water-treated seeds germinating for 2 days(2 biological repeats); GA2.1,GA2.2 GAs-treated seeds germinating for 2 days(2 biological repeats).

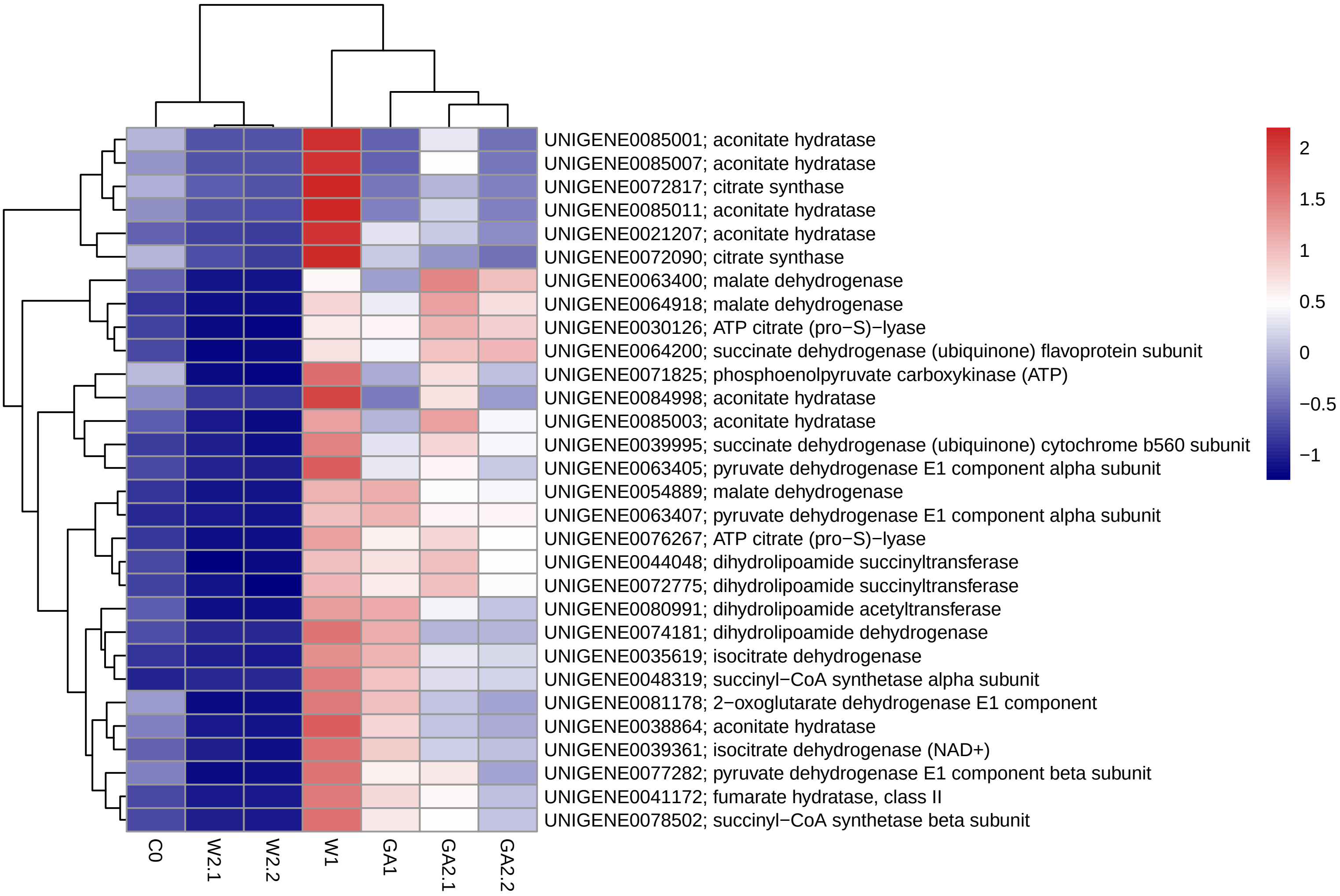


Figure S8. Heatmap diagram of expression levels for focused DEGs annotated in citrate cycle. The annotated unigenes are indicated at the side of each step. The sample names are showed at the bottom: C0 the seeds cold-stratificated for three months; W1 water-treated seeds germinating for 1 days; GA1 GAs-treated seeds germinating for 1 days; W2.1,W2.2 water-treated seeds germinating for 2 days(2 biological repeats); GA2.1,GA2.2 GAs-treated seeds germinating for 2 days(2 biological repeats).

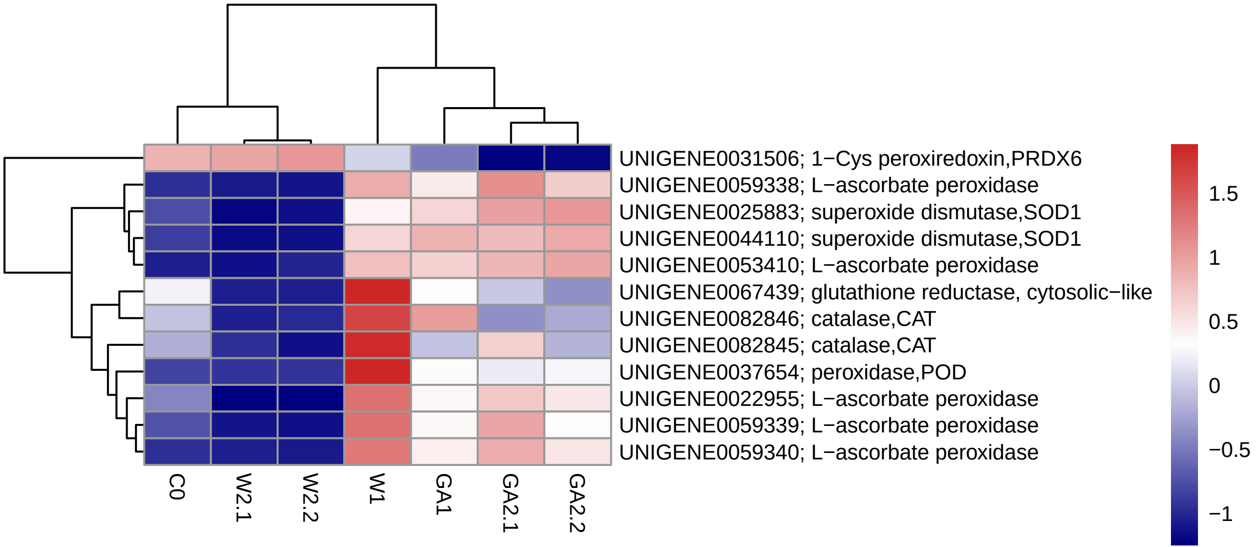


Figure S9. Heatmap diagram of expression levels for focused DEGs annotated in antioxidant enzymes. The annotated unigenes are indicated at the side of each step. The sample names are showed at the bottom: C0 the seeds cold-stratificated for three months; W1 water-treated seeds germinating for 1 days; GA1 GAs-treated seeds germinating for 1 days; W2.1,W2.2 water-treated seeds germinating for 2 days(2 biological repeats); GA2.1,GA2.2 GAs-treated seeds germinating for 2 days(2 biological repeats).

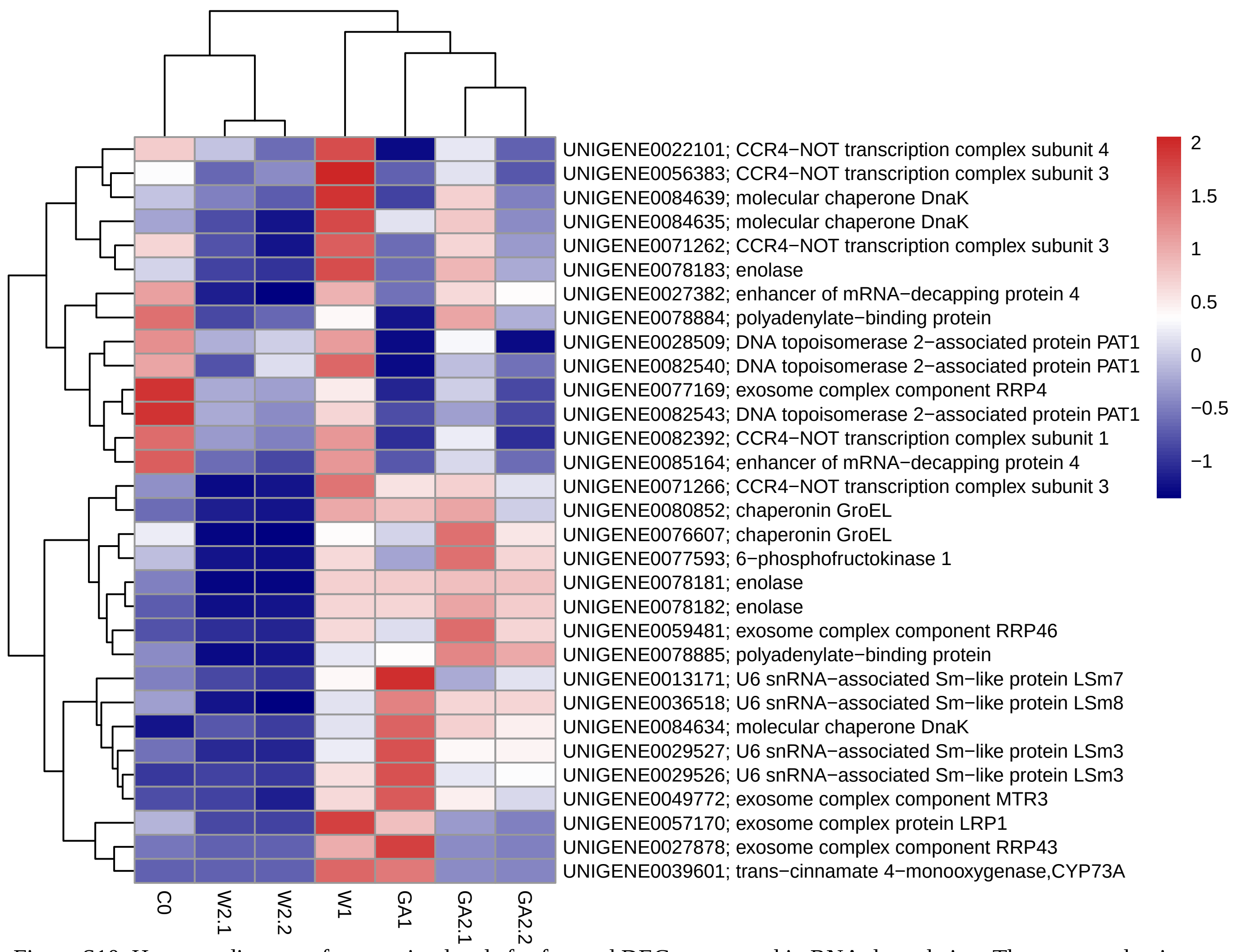


Figure S10. Heatmap diagram of expression levels for focused DEGs annotated in RNA degradation. The annotated unigenes are indicated at the side of each step. The sample names are showed at the bottom: C0 the seeds cold-stratificated for three months; W1 water-treated seeds germinating for 1 days; GA1 GAs-treated seeds germinating for 1 days; W2.1,W2.2 water-treated seeds germinating for 2 days(2 biological repeats); GA2.1,GA2.2 GAs-treated seeds germinating for 2 days(2 biological repeats).

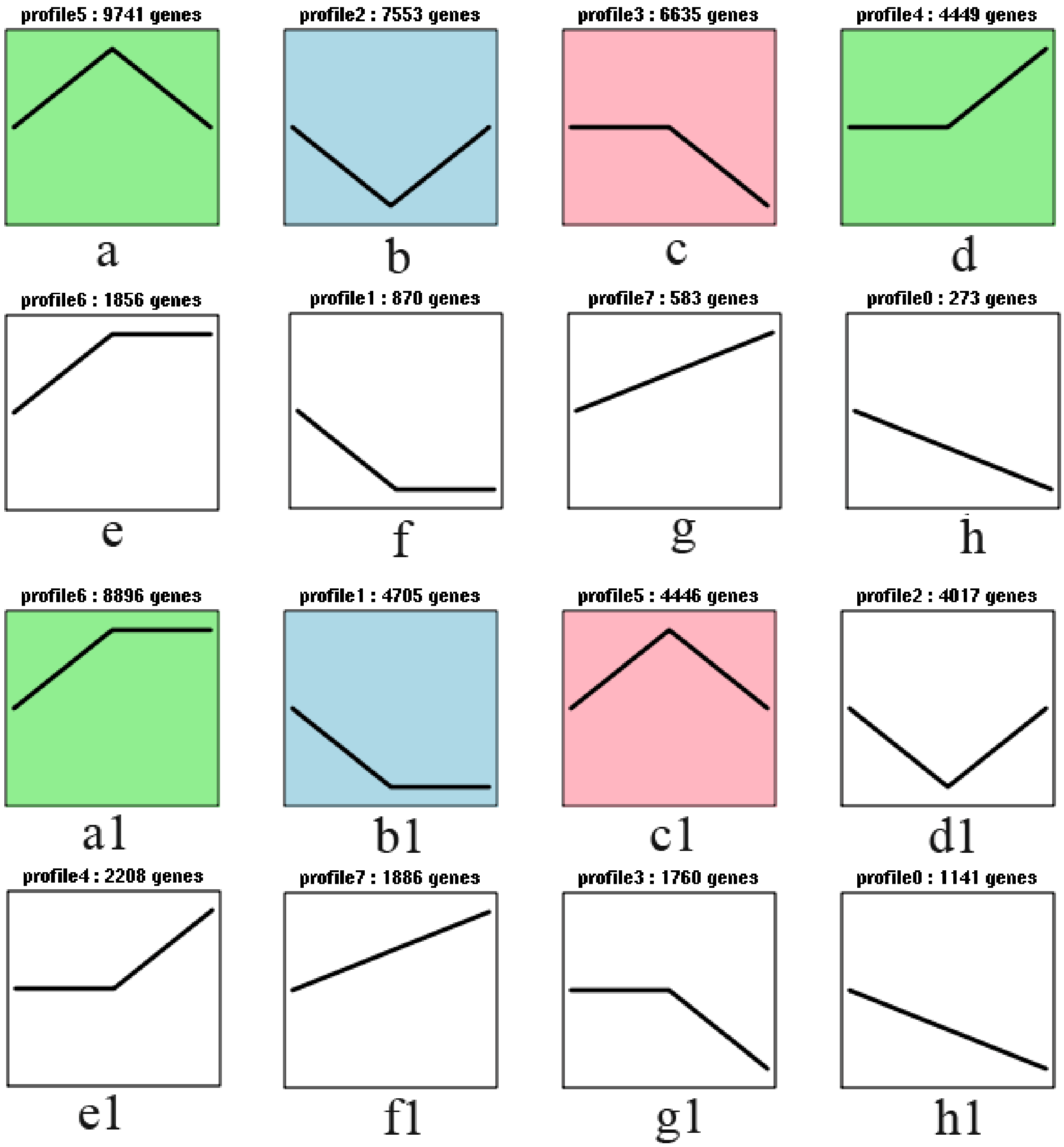


**Figure S11. Expression profiles of DEGs obtained from the STEM clustering.**

The 42,453 DEGs were classified into 8 clusters according to their expression patterns. **a-h** expression profiles of DEGs during germination of water-treated seeds. **a1-h1** expression profiles of DEGs during germination of GAs-treated seeds. Numbers indicated profiles or gene numbers. Significantly different profiles were represented by different background colors.

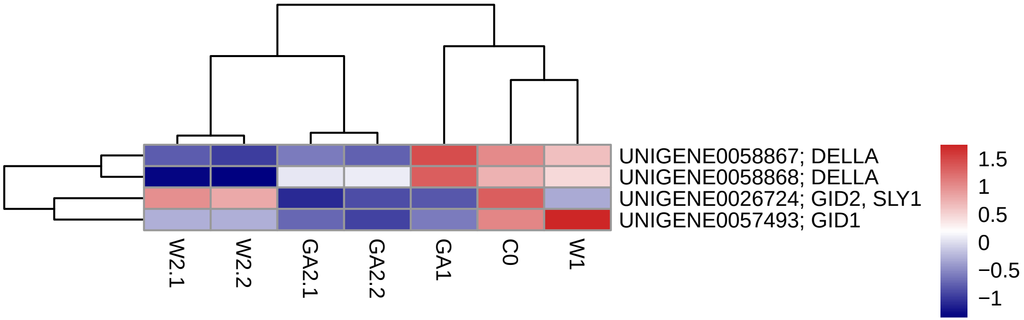


Figure S12 Heatmap diagram of expression levels for focused DEGs annotated in GAs signaling. The annotated unigenes are indicated at the side of each step. The sample names are showed at the bottom: C0 the seeds cold-stratificated for three months; W1 water-treated seeds germinating for 1 days; GA1 GAs-treated seeds germinating for 1 days; W2.1,W2.2 water-treated seeds germinating for 2 days(2 biological repeats); GA2.1,GA2.2 GAs-treated seeds germinating for 2 days(2 biological repeats).

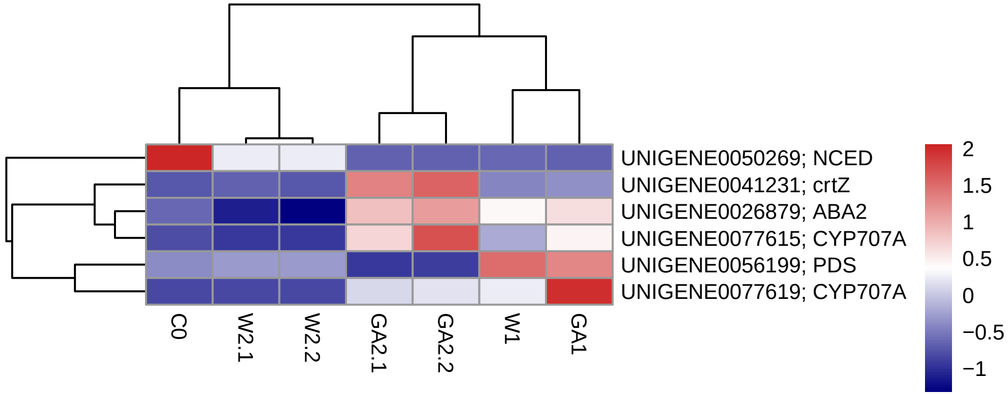


Figure S13. Heatmap diagram of expression levels for focused DEGs annotated in ABA biosynthesis. The annotated unigenes are indicated at the side of each step. The sample names are showed at the bottom: C0 the seeds cold-stratificated for three months; W1 water-treated seeds germinating for 1 days; GA1 GAs-treated seeds germinating for 1 days; W2.1,W2.2 water-treated seeds germinating for 2 days(2 biological repeats); GA2.1,GA2.2 GAs-treated seeds germinating for 2 days(2 biological repeats).

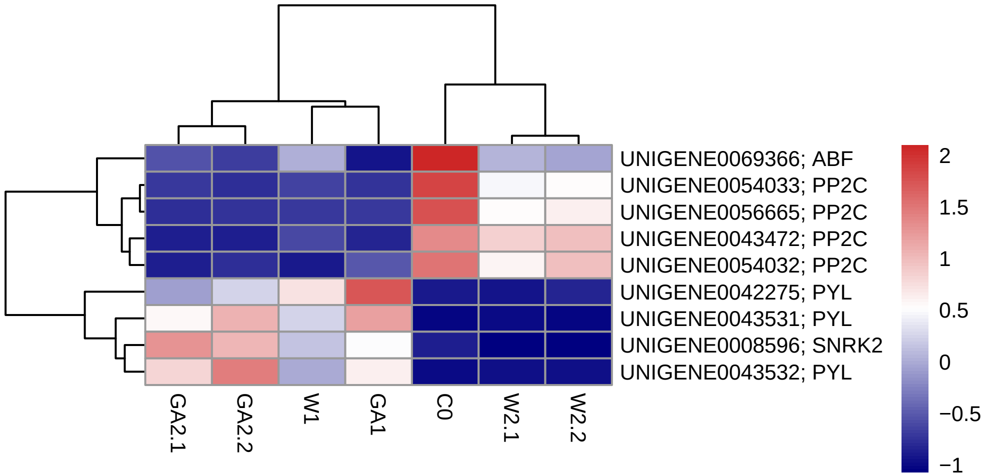


Figure S14. Heatmap diagram of expression levels for focused DEGs annotated in ABA signaling. The annotated unigenes are indicated at the side of each step. The sample names are showed at the bottom: C0 the seeds cold-stratificated for three months; W1 water-treated seeds germinating for 1 days; GA1 GAs-treated seeds germinating for 1 days; W2.1,W2.2 water-treated seeds germinating for 2 days(2 biological repeats); GA2.1,GA2.2 GAs-treated seeds germinating for 2 days(2 biological repeats).

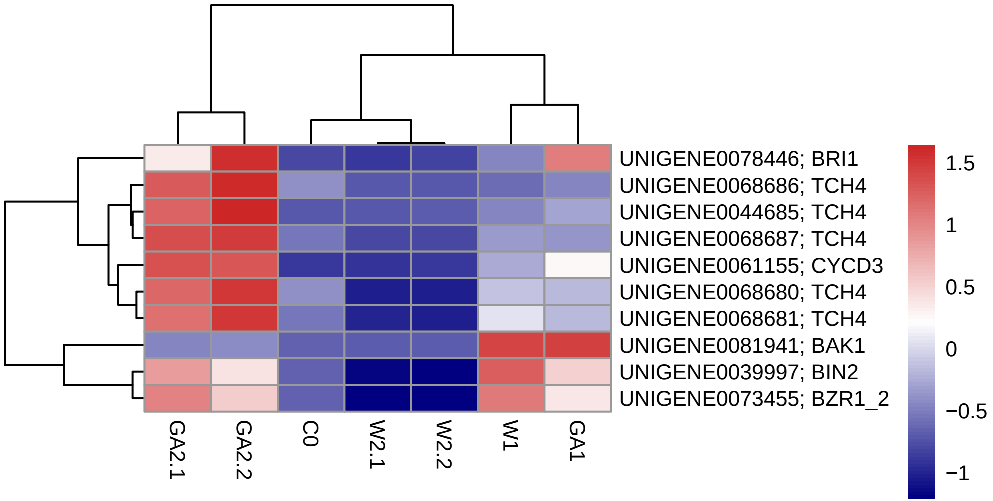


Figure S15. Heatmap diagram of expression levels for focused DEGs annotated in BRs signaling. The annotated unigenes are indicated at the side of each step. The sample names are showed at the bottom: C0 the seeds cold-stratificated for three months; W1 water-treated seeds germinating for 1 days; GA1 GAs-treated seeds germinating for 1 days; W2.1,W2.2 water-treated seeds germinating for 2 days(2 biological repeats); GA2.1,GA2.2 GAs-treated seeds germinating for 2 days(2 biological repeats).

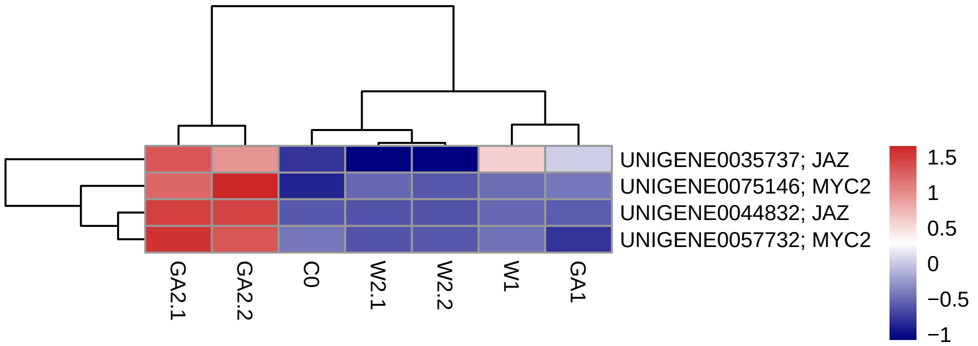


Figure S16. Heatmap diagram of expression levels for focused DEGs annotated in JAs signaling. The annotated unigenes are indicated at the side of each step. The sample names are showed at the bottom: C0 the seeds cold-stratificated for three months; W1 water-treated seeds germinating for 1 days; GA1 GAs-treated seeds germinating for 1 days; W2.1,W2.2 water-treated seeds germinating for 2 days(2 biological repeats); GA2.1,GA2.2 GAs-treated seeds germinating for 2 days(2 biological repeats).

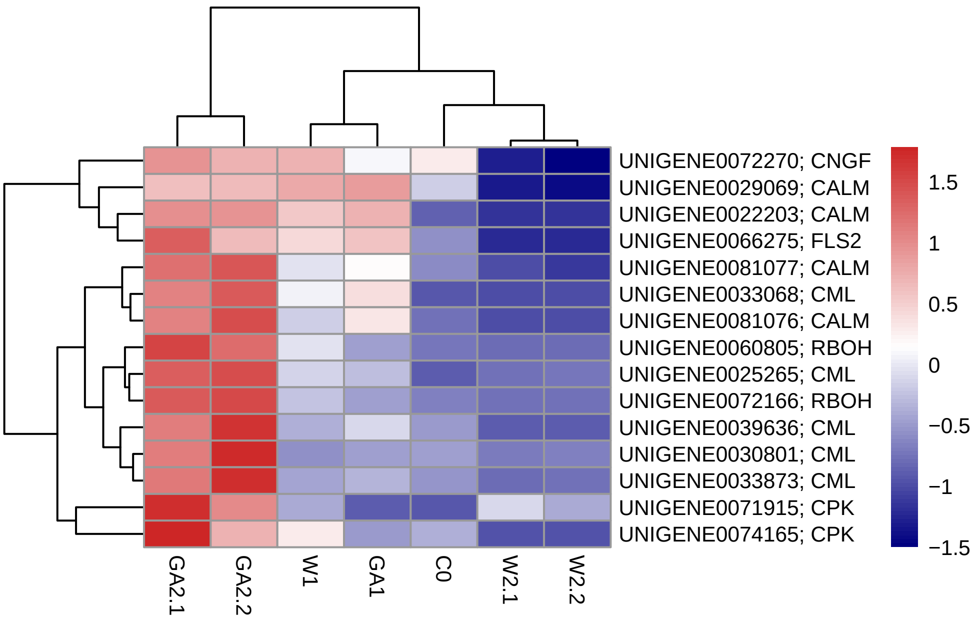


Figure S17. Heatmap diagram of expression levels for focused DEGs annotated in ROS signaling. The annotated unigenes are indicated at the side of each step. The sample names are showed at the bottom: C0 the seeds cold-stratificated for three months; W1 water-treated seeds germinating for 1 days; GA1 GAs-treated seeds germinating for 1 days; W2.1,W2.2 water-treated seeds germinating for 2 days(2 biological repeats); GA2.1,GA2.2 GAs-treated seeds germinating for 2 days(2 biological repeats).

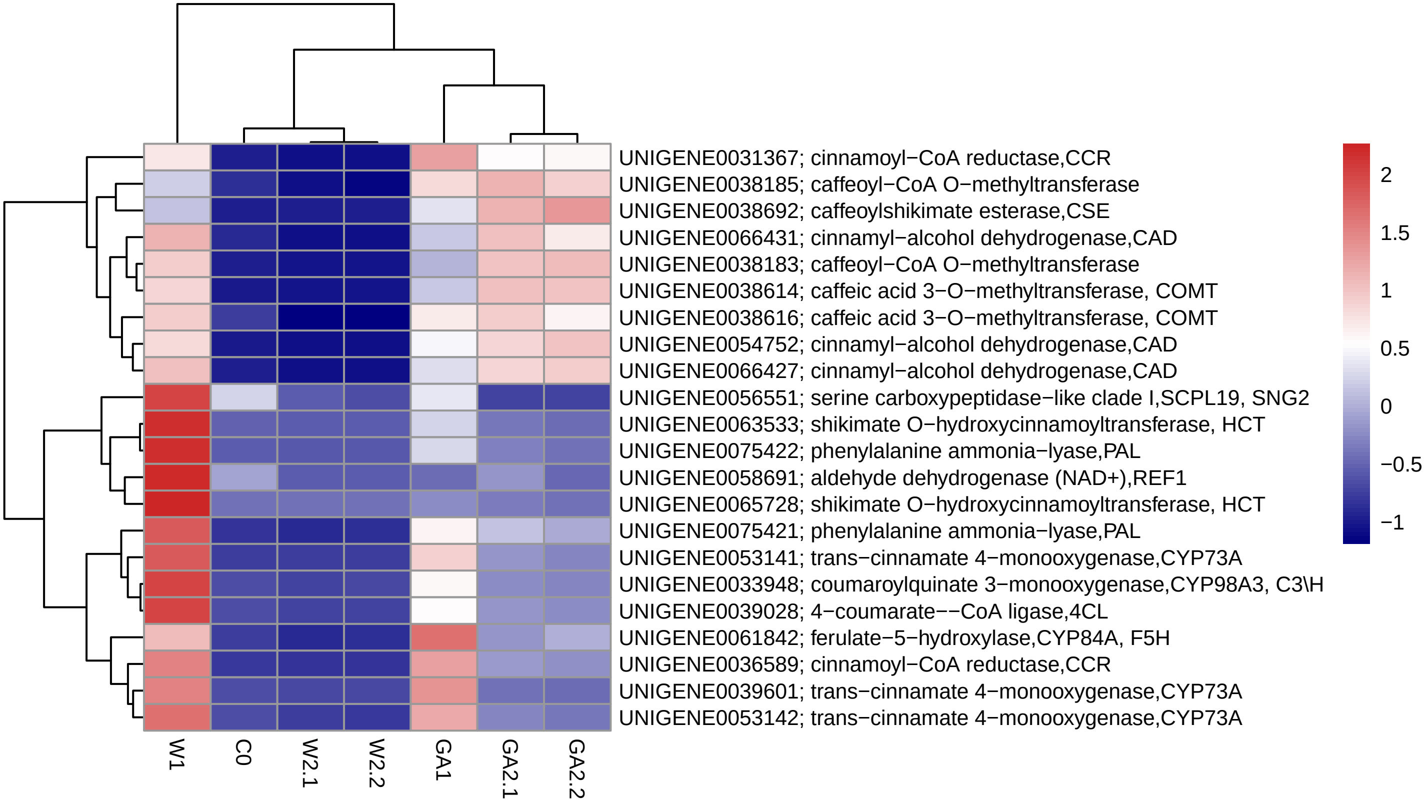


Figure S18. Heatmap diagram of expression levels for focused DEGs annotated in lignin biosynthesis. The annotated unigenes are indicated at the side of each step. The sample names are showed at the bottom: C0 the seeds cold-stratificated for three months; W1 water-treated seeds germinating for 1 days; GA1 GAs-treated seeds germinating for 1 days; W2.1,W2.2 water-treated seeds germinating for 2 days(2 biological repeats); GA2.1,GA2.2 GAs-treated seeds germinating for 2 days(2 biological repeats).

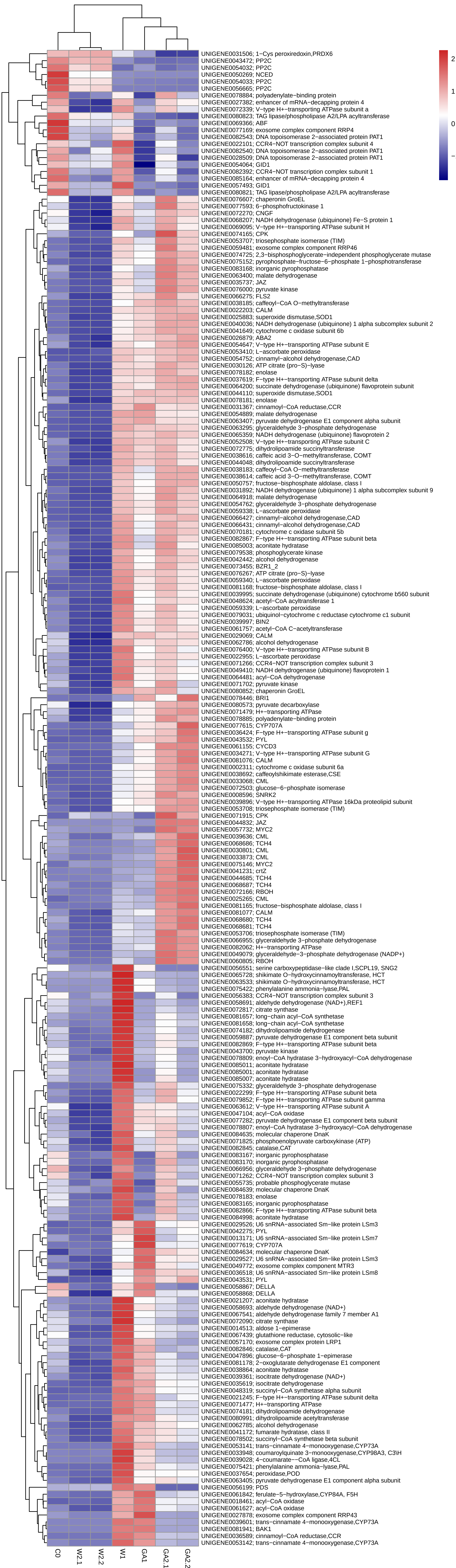


Figure S19. Heatmap diagram of expression levels for focused DEGs annotated in all aforementioned genes. The annotated unigenes are indicated at the side of each step. The sample names are showed at the bottom: C0 the seeds cold-stratificated for three months; W1 water-treated seeds germinating for 1 days; GA1 GAs-treated seeds germinating for 1 days; W2.1,W2.2 water-treated seeds germinating for 2 days(2 biological repeats); GA2.1,GA2.2 GAs-treated seeds germinating for 2 days(2 biological repeats).
